# Beyond detoxification: a role for mouse mEH in the hepatic metabolism of endogenous lipids

**DOI:** 10.1007/s00204-017-2060-4

**Published:** 2017-10-03

**Authors:** Anne Marowsky, Imke Meyer, Kira Erismann-Ebner, Giovanni Pellegrini, Nandkishor Mule, Michael Arand

**Affiliations:** 1 Institute of Pharmacology and Toxicology, University of Zurich, Winterthurerstrasse 190, 8057 Zurich, Switzerland; 2 Laboratory for Animal Model Pathology (LAMP), Institute of Veterinary Pathology, University of Zurich, Winterthurerstrasse 268, 8057 Zurich, Switzerland

**Keywords:** Microsomal epoxide hydrolase, Liver, EET, EpOME, Lipid signaling

## Abstract

Microsomal and soluble epoxide hydrolase (mEH and sEH) fulfill apparently distinct roles: Whereas mEH detoxifies xenobiotics, sEH hydrolyzes fatty acid (FA) signaling molecules and is thus implicated in a variety of physiological functions. These epoxy FAs comprise epoxyeicosatrienoic acids (EETs) and epoxy-octadecenoic acids (EpOMEs), which are formed by CYP epoxygenases from arachidonic acid (AA) and linoleic acid, respectively, and then are hydrolyzed to their respective diols, the so-called DHETs and DiHOMEs. Although EETs and EpOMEs are also substrates for mEH, its role in lipid signaling is considered minor due to lower abundance and activity relative to sEH. Surprisingly, we found that in plasma from mEH KO mice, hydrolysis rates for 8,9-EET and 9,10-EpOME were reduced by 50% compared to WT plasma. This strongly suggests that mEH contributes substantially to the turnover of these FA epoxides—despite kinetic parameters being in favor of sEH. Given the crucial role of liver in controlling plasma diol levels, we next studied the capacity of sEH and mEH KO liver microsomes to synthesize DHETs with varying concentrations of AA (1–30 μM) and NADPH. mEH-generated DHET levels were similar to the ones generated by sEH, when AA concentrations were low (1 μM) or epoxygenase activity was curbed by modulating NADPH. With increasing AA concentrations sEH became more dominant and with 30 μM AA produced twice the level of DHETs compared to mEH. Immunohistochemistry of C57BL/6 liver slices further revealed that mEH expression was more widespread than sEH expression. mEH immunoreactivity was detected in hepatocytes, Kupffer cells, endothelial cells, and bile duct epithelial cells, while sEH immunoreactivity was confined to hepatocytes and bile duct epithelial cells. Finally, transcriptome analysis of WT, mEH KO, and sEH KO liver was carried out to discern transcriptional changes associated with the loss of EH genes along the CYP-epoxygenase–EH axis. We found several prominent dysregulations occurring in a parallel manner in both KO livers: (a) gene expression of *Ephx1* (encoding for mEH protein) was increased 1.35-fold in sEH KO, while expression of *Ephx2* (encoding for sEH protein) was increased 1.4-fold in mEH KO liver; (b) *Cyp2c* genes, encoding for the predominant epoxygenases in mouse liver, were mostly dysregulated in the same manner in both sEH and mEH KO mice, showing that loss of either EH has a similar impact. Taken together, mEH appears to play a leading role in the hydrolysis of 8,9-EET and 9,10-EpOME and also contributes to the hydrolysis of other FA epoxides. It probably profits from its high affinity for FA epoxides under non-saturating conditions and its close physical proximity to CYP epoxygenases, and compensates its lower abundance by a more widespread expression, being the only EH present in several sEH-lacking cell types.

**Electronic supplementary material:**

The online version of this article (doi:10.1007/s00204-017-2060-4) contains supplementary material, which is available to authorized users.

## Introduction

Microsomal epoxide hydrolase (mEH) and soluble epoxide hydrolase (sEH) belong to the epoxide hydrolase (EH) family, which catalyzes the hydrolysis of epoxides. mEH and sEH were first characterized as detoxifying enzymes, because they can convert genotoxic epoxides to less harmful dihydrodiols (Oesch 1973; Ota and Hammock 1980). In particular, numerous in vitro and in vivo studies provide evidence that mEH is a crucial enzyme in xenobiotic detoxification with its substrates comprising epoxide intermediates from chemicals (e.g., styrene, 1,2-butadiene) and pharmaceuticals (e.g., phenytoin, carbamazepine, oprozomib) (Fretland and Omiecinski 2000; Wang et al. 2017). However, its role in the metabolism of endogenous lipid epoxides is less clear. Older studies suggested a potential function for mEH in steroid biosynthesis, because it is able to hydrolyze epoxy steroids (Vogel-Bindel et al. 1982) and participates in the antiestrogen binding site (Mesange et al. 1998). More recently, certain polymorphisms of the human mEH gene, *EPHX1,* have been associated with an increased risk to develop preeclampsia, a severe pregnancy complication, characterized by hypertension (Groten et al. 2014; Laasanen et al. 2002; Pinarbasi et al. 2007; Zusterzeel et al. 2001).

By contrast, a large body of evidence demonstrates the involvement of sEH in various physiological processes, which is due to the ability of sEH to efficiently hydrolyze endogenous FA epoxides such as the AA-derived epoxyeicosatrienoic acids (EETs) (Yu et al. 2000) and linoleic acid-derived epoxy-octadecenoic acids (EpOMEs, also termed leukotoxins) (Moghaddam et al. 1997). EETs are generated in a cytochrome P450 (CYP)-epoxygenase-mediated reaction, in which each of the four AA double bonds can be oxidized and thus four epoxide-regioisomers are formed (5,6-, 8,9-, 11,12-, and 14,15-EET). EETs are strong vasodilators in various vessel beds (Fisslthaler et al. 1999) and exert angiogenic (Michaelis and Fleming 2006), anti-inflammatory (Node et al. 1999), antinociceptive (Inceoglu et al. 2008), and tissue-regenerating and -protective effects (Panigrahy et al. 2013). Their diols (dihydroxyeicosatrienoic acids, DHETs) are generally regarded as being less active, and sEH-mediated hydrolysis is thus thought to control levels of active EETs. By contrast, diols derived from EpOMEs, termed dihydroxy-EpOMEs or DiHOMEs, are more potent than their parental epoxides and display pro-inflammatory properties. They cause edematous lung injury in mammals (Moghaddam et al. 1997) and possibly contribute to acute respiratory distress syndrome (Zheng et al. 2001). Although mEH is also capable of metabolizing EETs and EpOMEs (Decker et al. 2012; Marowsky et al. 2009, 2016), endogenous epoxy FA metabolism seems clearly dominated by sEH due to its higher activity relative to mEH (Kodani and Hammock 2015; Spector and Kim 2015). Based on the maximal velocity Vmax, which indicates how many moles of epoxide are metabolized per gram enzyme in a minute under saturating conditions, human sEH is 170 times faster than mEH in hydrolyzing 14,15-EET (Decker et al. 2012), and mouse sEH is even 800 times faster than mEH with the same substrate (Marowsky et al. 2009). sEH and mEH are also distinct in their preference profiles, as human and mouse sEH show highest catalytic efficiencies (in descending order) with 14,15-EET > 9,10-EpOME > 12,13-EpOME > 11,12-EET ≫ 8,9-EET (Morisseau et al. 2010; Newman et al. 2002). Available data for mEH are limited to EETs, and indicate an inverse preference profile relative to sEH, as human and mouse mEH show highest catalytic efficacies with 11,12-EET > 8,9-EET > 14,15-EET (Decker et al. 2012; Marowsky et al. 2009).

In line with the major role of sEH in EET hydrolysis, inhibition of sEH or genetic deletion of *Ephx2*, the gene encoding for sEH, results in increased EET levels and modifications of various EET-mediated physiological effects including blood pressure (Sinal et al. 2000; Sun et al. 2014), inflammation (Bettaieb et al. 2014; Kim et al. 2015; Tao et al. 2016), and inflammatory pain (Inceoglu et al. 2006). In contrast to sEH inhibitors, which elaborated over recent years (Morisseau et al. 1999; Ulu et al. 2012), the available mEH inhibitors have the disadvantage that they are either highly unstable [2-nonylsulfanyl-propanamide (NSPA) and elaidamide each contain an amide group and are readily metabolized by amide hydrolases (Morisseau et al. 2008)] or highly toxic (1,1,1-trichloropropene oxide, TCPO), which prevents their application in vivo. In absence of stable and well-tolerated mEH inhibitors, mEH KO mice provide a useful tool to assess the physiological importance of mEH. Yet mEH KO mice lack an obvious phenotype (Miyata et al. 1999) and mEH KO characterization has so far focused exclusively on aberrant effects in xenobiotic metabolism with compounds such as styrene (Carlson 2010), DMBA (Miyata et al. 1999; Rajapaksa et al. 2007), and benzene (Bauer et al. 2003). Here, we expand on the phenotypic characterization of mEH KO mice by focusing on changes in endogenous epoxy FA metabolism, using plasma and liver homogenates as physiological samples. These data were correlated with liver transcriptome data from WT control, sEH KO, and mEH KO mice with focus on differentially expressed genes underlying the CYP epoxygenase–epoxide hydrolase system. Finally, the hepatic expression pattern of mEH and sEH protein expression was investigated.

## Materials and methods

sEH KO (Sinal et al. 2000) and mEH KO mice (Miyata et al. 1999) were kindly provided by Dr. F. J. Gonzalez (NIH, Bethesda, MD, USA). Both KO lines were backcrossed for 7–9 generation onto C57BL/6 background prior to this study and preliminary experiments were conducted with WT littermates from both lines. Since no difference was detected between sEH and mEH WT littermates in CYP-epoxygenases/EH-dependent metabolism, WT mice from both lines were used for all experiments presented in the current study. All mice were housed in single-ventilated cages in facilities, which met the criteria for specific pathogen conditions (SPF); animals were kept at a standard 12:12 h light cycle and had ad libitum access to water and standard chow. Except for immunohistochemical experiments, exclusively male mice (8–16 weeks of age) were used for this study. For immunohistochemistry, liver slices were obtained from both male and female mice (8–16 weeks of age). All procedures were approved by the local veterinary authorities and were performed in accordance with the European Community Council Directive (86/609/EEC). Experiments were designed to minimize the number of animals used and their suffering, according to the guidelines of the Swiss Academy of Medical Sciences.

### Genotyping

mEH KO, sEH KO, and WT genotype were confirmed by standard polymerase chain reaction (PCR), using specific primer pairs. Genomic DNA was extracted from mouse ear biopsies and the following primers were used to identify homozygous WT and mEH KO mice: mKO forward 5′-CCCGGGACAAGGAGGAGACC-3′ and mKO reverse 5′-AAGGATCACAGGGTGAAAGGAA-3′. The PCR product for homozygous WT had a size of 800 bp and that for homozygous mEH KO mice a size of 1400 bp. For genotyping sEH KO mice, the primers sKO forward 5′-GCGAGGGGCGGTGCTGAGATTGG-3′ and sKO reverse 5′-CTGGAAAGCAATTTGAAACCTGGG-3′ were used. The PCR product for homozygous WT had a size of 305 bp, for homozygous sEH KO a size of 1444 bp.

### Plasma and liver oxylipin profiles

Animals were killed by i.p. injection of 160–200 μl Sodium-Pentobarbital (50 mg/ml). Blood was immediately taken from V. cava, using a syringe that was rinsed before with 10% EDTA (pH 8). The collected blood was centrifuged for 10 min at 1700×*g* in a benchtop centrifuge (Eppendorf 5417R); plasma was removed and frozen at −20 °C after adding the antioxidant butylated hydroxytoluene. For oxylipin extraction, 150 μl of plasma was added to 150 μl acetonitrile/methanol (1:1), including the deuterated standards for 8,9-, 11,12-, 14,15-EETs and DHETs; 9,10-, and 12,13-EpOMEs; and 5-, 8-, and 12-HETEs. After addition of 700 μl phosphate-buffered saline (PBS, pH 7.4), the samples were centrifuged (5 min, 10,000×*g*) and subjected to solid-phase extraction, using ISOLUTE^®^ C18 solid-phase extraction columns (100 mg sorbent mass; Biotage, Uppsala, Sweden). The columns were primed with 2 × 1 ml acetonitrile and 2 × 1 ml PBS containing 5% methanol (pH 7.4). After loading the samples, columns were washed with 1 ml PBS containing 5% methanol, dried, and eluted with 3 × 600 μl ethyl acetate. The eluate was dried under nitrogen, and extracted lipids were re-dissolved in 100 μl methanol/20 mM Tris, pH 7.4 (1:1), and subjected to liquid chromatography-tandem mass spectrometry (LC–MS/MS). Incubation of liver homogenates with 50 μM AA was carried out as described previously (Marowsky et al. 2016).

### Preparation of hepatic microsomal and cytosolic fractions

100–200 mg frozen livers of all three genotypes (WT, mEH KO, sEH KO) were homogenized on ice in 1 ml buffer containing 125 mM KCl, 250 mM sucrose, 100 mM potassium phosphate, and 1 mM EDTA (pH 7.4). The samples were centrifuged (5 min at 600×*g*; 20 min at 9000×*g*); the supernatant (S9 fraction) was removed and further centrifuged for 20 min at 100,000×*g* in an ultracentrifuge, using a TLA-100.3 rotor (Beckman Coulter, Brea, CA, USA), which yielded the cytosolic fraction as supernatant. The pellet, containing the microsomal fraction, was washed in 500 µl Tris buffer (0.1 M Tris, 0.125 M KCl, pH 7.4), ultra-centrifuged again for 20 min at 100,000×*g*, and resuspended in Tris buffer. The final microsomes were homogenized and shock-frozen in liquid nitrogen. The total protein content of microsomal and cytosolic fractions was determined using a standard Bradford protein assay.

### Turnover assays with EETs and selective EH inhibitors

Cytosolic (0.2–0.3 μg total protein) or microsomal fractions (3–10 μg total protein) were added to a final volume of 50 μl of Tris buffer (10 mM Tris, 100 mM NaCl, 1 mM EDTA, pH 7.4) and preincubated on ice for 10 min with and without the selective sEH inhibitor trans-4-[4-(3-adamantan-1-y1-ureido)-cyclohexyloxy]-benzoic acid (tAUCB; 1 μM) or a combination of the mEH inhibitors 1,1,1-trichloropropene oxide (TCPO; 1 mM), and 2-nonylsulfanyl-propanamide (NSPA; 10 μM) (Morisseau et al. 2008). The reactions were started by adding 10 μM 14,15-EET as substrate to cytosolic fractions or 2–4 μM 11,12-EET to microsomes, and samples were incubated for 10 min at 37 °C. The reactions were stopped by adding 50 µl acetonitrile and subsequently 100 µl ddH_2_O containing the deuterated standards for the respective EETs and DHETs. Samples were centrifuged and analyzed without extraction by LC–MS/MS.

### Epoxygenase-epoxide hydrolase activity assay with microsomes

Microsomes (containing 50 μg protein) were added to a final vol of 50 μl buffer (50 mM Tris, pH 7.4, 10 mM MgCl_2_, 150 mM KCl, and 8 mM sodium isocitrate). The reaction was started by adding 2 mM NADPH, 0.6 U/mL isocitrate dehydrogenase Type IV, and AA. Samples were incubated at 37 °C for 10–30 min. The reactions were stopped with 100 μl ethyl acetate containing deuterated standards. The organic phase was removed and samples were extracted a second time with 100 μl ethyl acetate. Organic phases were combined and dried under nitrogen. Lipids were re-dissolved in Tris (20 mM, pH 7.4) and analyzed by LC–MS/MS.

### Western blot analysis

Frozen liver samples (200 mg) from WT and mEH KO mice were homogenized in 10 vol RIPA lysis buffer (20 mM Tris, 150 mM NaCl, 1% Triton X-100, 1% Na-deoxycholate, 0.1% SDS, 1 Protease inhibitor cocktail tablet (Complete Mini, Roche, Basel, Switzerland)), and centrifuged in a benchtop centrifuge for 10 min at 13,000×*g*. The cleared sample was assayed for total protein content using the Bradford method. Samples (20 μg of protein per lane) were separated on a 12.5% SDS-PAGE and transferred onto PVDF membranes (Immobilon-P, Millipore, Billerica, MA, USA) using the semi-dry Trans-Blot^®^Turbo™ Transfer Starter System (BioRad, Hercules, CA, USA). The primary antibodies used were raised in rabbits against rat sEH [in-house (Marowsky et al. 2009)] and against mouse GAPDH (Sigma-Aldrich, St. Louis, MO, USA), each applied in dilutions of 1:10,000. The secondary antibody used was raised in donkey against rabbit IgG and fluorescent-labeled (1:8000, IRDye^®^ 800CW Donkey anti-Rabbit IgG, Li-cor Biosciences, Lincoln, NE, USA). Fluorescence of immunoreactive bands was detected at 800 nm with an Odyssey IR imager (Li-cor Biosciences, Lincoln, NE, USA), followed by quantification of band intensities with Odyssey IR imager software (Image Studio 3.1).

### Histology

Animals were euthanized by CO_2_ asphyxiation. The liver was quickly removed and fixed in 4% neutral-buffered formalin (Formafix, Hittnau, Switzerland). Sections of the left lateral and median lobes were dehydrated through graded alcohols and embedded in paraffin wax. Consecutive sections (3–5 µm) were prepared, mounted on glass slides, and subjected to immunohistochemistry (IHC). IHC was performed using rabbit anti-mEH (directed against a mixture of rat, mouse, and human mEH protein, made in-house) and rabbit anti-sEH antibody (Marowsky et al. 2009). Briefly, sections were deparaffinised in xylene and rehydrated in decreasing concentrations of ethanol. Sections underwent antigen retrieval by incubation with 10 mM citrate buffer (pH 6.0). For mEH IHC, slides were incubated for 1 h at 37 °C with the primary antisera (1:2000, diluted in Dako antibody diluent, Dako-Agilent Technologies, Denmark), whilst incubation with the anti-sEH antibody (1:1500) occurred overnight at 4 °C. In both staining procedures, this was followed by incubation for 30 min with a horse radish peroxidase (HRP)-labeled polymer, conjugated to a secondary anti-rabbit antibody (Dako EnvisionTM System, Dako-Agilent Technologies). The reaction was visualized using 3,3′-diaminobenzidine (DAB) as chromogen, followed by light counterstain with hematoxylin. After rinsing, slices were dehydrated in ascending concentrations of ethanol, cleared with xylene, cover-slipped, and mounted. All immunohistological stainings were performed using an Autostainer (Dako Autostainer Universal Staining System Model LV-1, Dako-Agilent Technologies). Slides were scanned using a digital slide scanner (NanoZoomer-XR C12000; Hamamatsu, Japan) and pictures were taken using the NDP.view2 viewing Software (U12388-01, Hamamatsu).

### LC–MS/MS analysis of oxylipins

Analysis of eicosanoid profiles was performed as established previously (Marowsky et al. 2016). 2.5 ng of each of the following compounds served as standard for quantification: 8,9-EET, 11,12-EET, 14,15-EET, 5,6-DHET, 8,9-DHET,11,12-DHET, 14,15-DHET, 9,10-EpOME, 12,13-EpOME, 5-HETE, 8-HETE, 12-HETE, 20-HETE, AA, 8,9-EET-*d11*, 11,12-EET-*d11*, 14,15-EET-*d11*, 8,9-DHET-*d11*, 11,12-DHET-*d11*, 14,15-DHET-*d11*, 5-HETE-*d8*, 12-HETE-*d8*, 20-HETE-*d6*, AA-*d8*. Deuterated standards, 3 ng of each, were added to each experimental sample and served as internal standards. Quantification was done by determining the area under curve (AUC) using MultiQuant Software 2.1.1 (Sciex, USA). Values were corrected for extraction efficiency and change in MS sensitivity by using the internal standard peak area. For verification, a sample containing 250 pmol of epoxides (EETs or EpOMEs) in Tris buffer (20 mM, pH 7.4) was injected in parallel to a sample, in which 250 pmol epoxide was completely metabolized by recombinant sEH before. The AUC for the diol was then assumed to correspond to 250 pmol and values were accordingly calculated.

### Liver transcriptome analysis

Total RNA from sEH KO, mEH KO, and WT liver (*n* = 3 for each) was extracted using a commercially available isolation kit. All following steps (purification of mRNA, transcription into cDNA and generation of a library) were carried out by the Functional Genomic Center of the University of Zurich (FGCZ), following the TruSeq Stranded mRNA sample protocol from Illumina (San Diego, Ca, USA). Bioinformatic analysis was performed using the R package DezRun (https://github.com/uzh/ezRun) within the data analysis framework SUSHI (Hatakeyama et al. 2016). The raw reads were quality-checked using FastQC (http://www.bioinformatics.babraham.ac.uk/projects/fastqc/) and FastQ Screen (http://www.bioinformatics.babraham.ac.uk/projects/fastq_screen/). Quality controlled reads (adaptor trimmed, first 3 bases hard-trimmed, minimum tail quality Q20, minimum read length 20 nt) were aligned to the reference genome (Ensembl GRCm38.p3) using the STAR aligner. Expression counts were computed using countOverlaps function in the Bioconductor package GenomicRanges. Differential expression analysis was performed using the edgeR package with raw read counts normalized using the trimmed mean of *M* values method; differential expression was computed using the Generalized Linear Mode likelihood ratio test. Project accession number: PRJEB21957.

## Materials

All fatty acids used in this study were purchased without exception from Cayman Chemicals, Ann Arbor, MI, USA. TCPO was purchased from EGA-Chemie (Steinheim, Germany). tAUCB and NSPA were kind gifts from Christophe Morisseau, University of California, Davis, CA, USA.

## Results

### Fatty acid epoxide:diol ratios in plasma of WT, mEH KO, and sEH KO animals

**Fig. 1:**
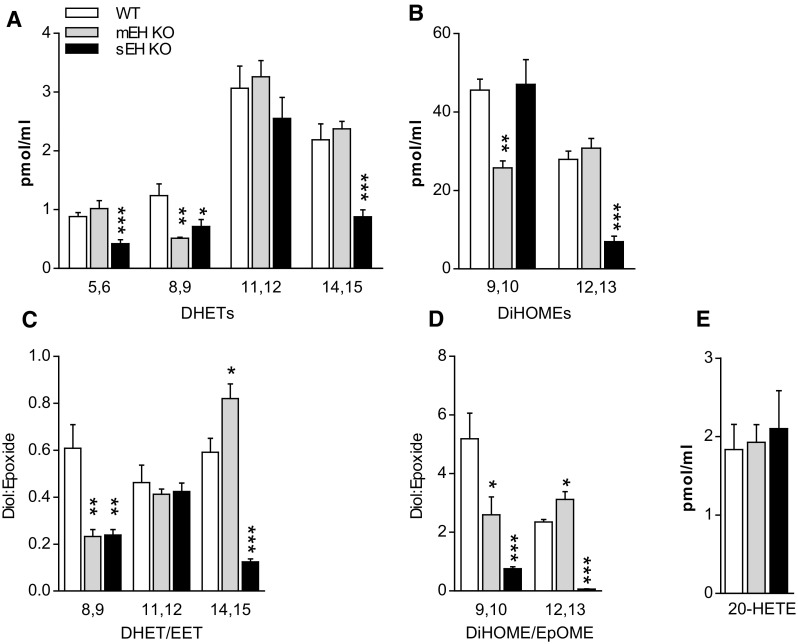
Plasma diol and 20-HETE levels and diol:epoxide ratios in WT control, mEH KO, and sEH KO mice. **a** Levels for DHETs, the secondary metabolites from AA generated by EH conversion; 8,9-DHET levels were significantly lower in mEH KO relative to WT mice. **b** Levels for DiHOMEs, the secondary metabolites from linoleic acid generated by EH conversion; 9,10-DiHOME levels were significantly lower in mEH KO relative to WT mice. **c** The DHET:EET ratio, reflecting the hydrolysis rate, was lower for 8,9-, but higher for 14,15-EET in mEH KO compared to WT plasma. **d** The DiHOME:EpOME ratio was reduced for 9,10-EpOME, but increased for 12,13-EpOME in mEH KO relative to WT plasma. **e** 20-HETE levels were similar across genotypes. 1-way ANOVA followed by Dunnett’s post tests; statistically significant differences for WT vs KO are indicated. * *p* < 0.05, ** *p* < 0.01, *** *p* < 0.001

To test for a possible impact of mEH on epoxide FA metabolism, we compared WT and mEH KO oxylipin profiles in plasma; in addition, the sEH KO plasma profile was assessed. LC–MS/MS analysis encompassed AA-derived EETs, linoleic acid-derived 9,10- and 12,13-EpOMEs as well as the respective EH metabolites, namely DHETs and DiHOMEs. Furthermore, AA-derived monohydroxy metabolites (HETEs) were determined to elucidate possible secondary effects associated with mEH deficiency. All analytes were detectable in appreciable amounts. Compared to WT diol levels, mEH KO had significantly lower 8,9-DHET and 9,10-DiHOME levels (Fig. 1a, b). Importantly, diol:epoxide ratios (reflecting hydrolysis rates) for 8,9-DHET:EET and 9,10-DiHOME: EpOME (Fig. 1c, d) were also significantly reduced, suggesting that mEH plays an important role in the hydrolysis of 8,9-EET and 9,10-EpOME in vivo. By contrast, ratios for 14,15-DHET:EET and 12,13-DiHOME:EpOME were significantly increased in mEH KO relative to WT plasma, an indication for increased rather than decreased EH activity. Levels of 5-, 8-, 12-, 15-, and 20-HETE were similar in WT, mEH KO, and sEH KO plasma (Fig. 1e) (for detailed data and statistics see Suppl. Table 1). Results in this and the following figures are shown as average values ± SEM (standard error of the mean).

Plasma EET levels were reported to be largely controlled by hepatic sEH activity (Schuck et al. 2014). Thus, we hypothesized that increased hydrolysis of the sEH-preferred substrate 14,15-EET could be attributable to modified hepatic sEH activity in mEH KO liver. To investigate this further, we determined sEH activity and protein expression in mEH KO and WT liver.

### sEH activity and protein expression levels in mEH KO and WT control liver

**Fig. 2:**
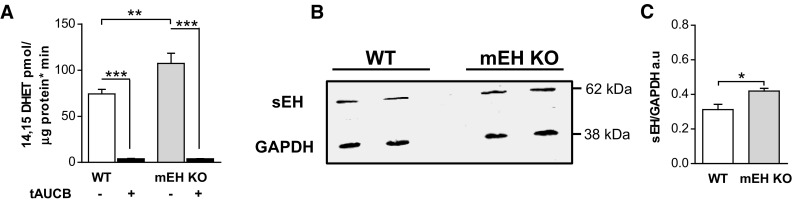
sEH activity and protein expression are increased in mEH KO compared to WT liver. **a** Turnover assay with WT and mEH KO liver cytosol using 14,15-EET as substrate in presence and absence of the selective sEH inhibitor tAUCB. The conversion rate to 14,15-DHET was 1.4-fold higher in mEH KO compared to WT cytosol (*n* = 5; 1-way ANOVA followed by Bonferroni post tests). **b** Representative immunoblot comparing sEH expression in WT and mEH KO liver. **c** Quantification of immunoreactive bands revealed a 1.3-fold higher sEH expression in mEH KO compared to WT liver (*n* = 3; unpaired Student’s *t* test). * *p* < 0.05, ** *p* < 0.01, *** *p* < 0.001

Cytosolic fractions from WT and mEH KO liver were prepared and hydrolysis rates with 14,15-EET as substrate were determined in the absence and the presence of the sEH inhibitor tAUCB (1 μM). The formation rate of 14,15-DHET in mEH KO cytosol was significantly higher (1.4-fold) than in WT; in both genotypes, formation of 14,15-DHET could almost completely be blocked (97%) by the sEH-specific inhibitor tAUCB (Fig. 2a). Furthermore, immunoblotting WT and mEH KO liver with a specific sEH antibody revealed that sEH protein expression was 1.3-fold higher in mEH KO compared to WT (Fig. 2b, c). Thus, sEH expression and activity are significantly increased in mEH KO relative to WT liver.

### Oxylipin profiles of WT, mEH KO, and sEH KO liver

**Fig. 3:**
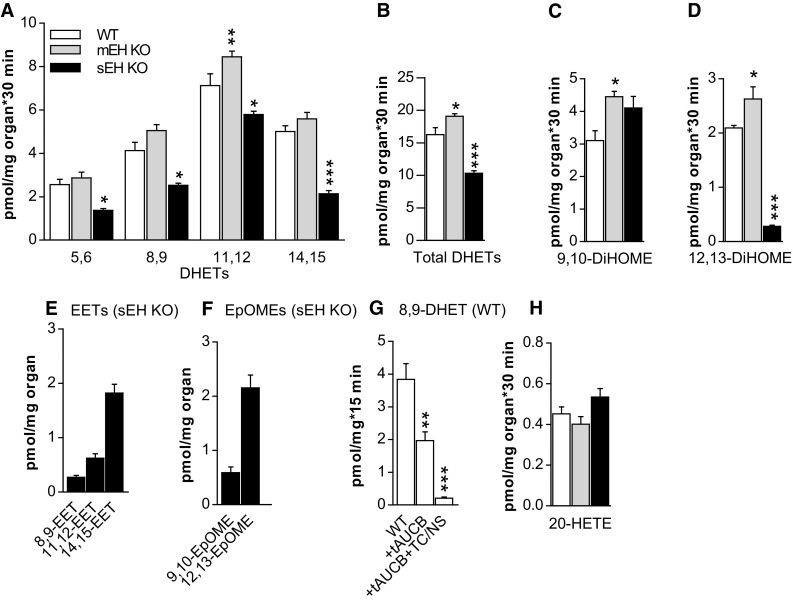
EH-mediated generation of FA diols in WT, mEH KO, and sEH KO liver homogenates under saturating conditions. **a** The formation rates for the four DHET regioisomers were generally higher in mEH KO, but lower in sEH KO compared to WT (statistical significances are indicated for WT vs KO; *n* = 5 for each genotype; 2-way ANOVA, followed by Dunnett’s post tests). **b** The formation rates for total DHETs, comprising 8,9-, 11,12-, and 14,15-DHET. In sEH KO liver, mEH could not entirely compensate for sEH loss, but still generated 60% of DHETs formed in WT liver by both EHs. **c**, **d** Formation rates for 9,10- and 12,13-DiHOMEs, which are higher in mEH KO compared to WT. 12,13-DiHOME generation was significantly reduced in sEH KO, pointing towards a pivotal role for sEH in its formation. **e** EETs and **f** EpOMEs were exclusively detectable in sEH KO, but not in WT and mEH KO liver homogenates after 30 min incubation with AA. **g** Turnover of 8,9-EET with WT liver without inhibitors and in presence of the sEH inhibitor tAUCB alone and in combination with the mEH inhibitors TCPO/NSPA (TC/NS). DHET formation was reduced by 50% in presence of tAUCB and by 95% in presence of all blockers, supporting the notion that mEH and sEH are the only EET-conversing EHs in mouse liver. **h** 20-HETE formation was similar across genotypes. *n* = 5 for each genotype and metabolite or treatment group; for **b**–**d**, **g**, **h** 1-way ANOVA followed by Dunnett’s post test. * *p* < 0.05, ** *p* < 0.01, *** *p* < 0.001

Given the unexpected upregulation of sEH in mEH KO liver, we next assessed the genotype-specific capacity to generate epoxygenase- and EH-mediated products in liver. sEH KO, mEH KO and WT liver homogenates were incubated with a high concentration of AA (50 μM) in the presence of a NADPH-regenerating system, and EET, DHET, EpOME, and DiHOME formation rates were analyzed. For each metabolite, the respective background level was determined in liver homogenates without addition of AA; these levels were then subtracted for the calculation of the final formation rate. Under these conditions, EET and EpOME levels were only detectable in absence of sEH, underlining the high capacity of the enzyme. To ascertain that the only EHs present in C57BL/6 WT liver are mEH and sEH, we incubated WT liver with a mixture containing 2 µM of each 8,9-, 11,12-, and 14,15-EET in absence and presence of the sEH inhibitor tAUCB alone (1 µM) and tAUCB (1 µM) in combination with the mEH inhibitors TCPO (1 mM) and NSPA (10 µM). tAUCB blocked 50% of the turnover to 8,9-DHET, tAUCB and TCPO/NSPA together 95% (Fig. 3g) with the residual turnover probably due to incomplete block by tAUCB as observed earlier in Fig. 2a. Similar results were obtained for 11,12- and 14,15-EET (data not shown). These findings indicated that with high probability no EH other than mEH and sEH are expressed in WT liver. In sEH KO liver, mEH is with high probability the only EH present and thus responsible for total FA epoxide hydrolysis (see next paragraph and discussion). The mEH-mediated diol formation in sEH KO liver was substantial and reached 61% for 8,9-DHET, 80% for 11,12-DHET, and 43% for 14,15-DHET relative to WT (Fig. 3a). Surprisingly, the formation rate of 9,10-DiHOME was even higher in sEH KO than in WT (134%), while the 12,13-DiHOME formation rate only reached 14% of the one in WT (Fig. 3c, d). With regard to HETEs, the formation rates were the same for all genotypes including the one for the vasoconstrictor 20-HETE (Fig. 3h, Suppl. Table 2).

**Fig. 4:**
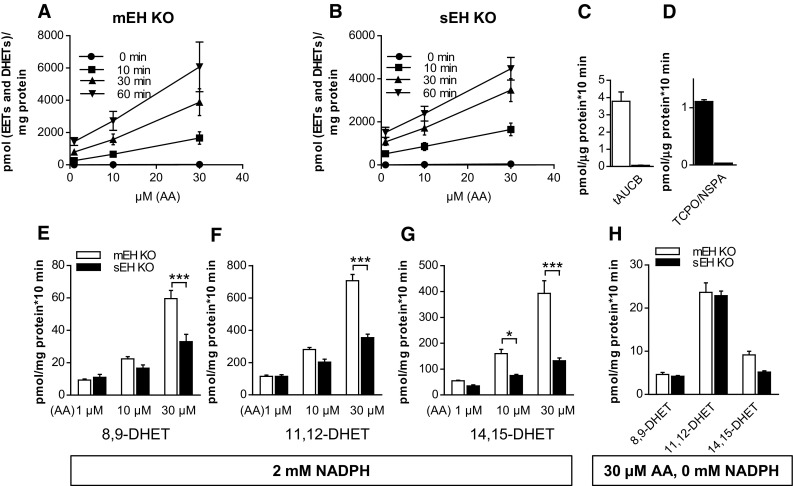
mEH contribution to DHET formation depends on the availability of AA and regenerating system. **a** In mEH KO liver microsomes formation rate of [EETs and DHETs], assumed to reflect epoxygenase activity, increased steadily with rising AA concentrations and incubation time, indicating that hepatic epoxygenases are not saturated under these conditions. **b** The same as **a** for sEH KO microsomes (*n* = 3 for **a**, **b**). **c** 11,12-DHET formation rate in mEH KO liver microsomes in absence and presence of the sEH inhibitor tAUCB. tAUCB blocked 98% of 11,12-DHET formation. **d** The same as **c** with sEH KO microsomes and the mEH inhibitors TCPO/NSPA, which blocked 97% of the DHET formation. **e**–**g** Liver microsomes were incubated with AA concentrations varying from 1 to 30 µM in the presence of a regenerating system and 2 mM NADPH. With lower AA concentrations, DHET formation rates were similar between genotypes for the mEH-preferred EET regioisomers (8,9 and 11,12), but were significantly distinct with high AA concentration. **h** DHET formation rates were similar between the two genotypes even with 30 μM AA, if the regenerating system including NADPH was omitted (*n* = 4, 2-way ANOVA with Bonferroni post tests). * *p* < 0.05, *** *p* < 0.001

Since in most studies an excess of AA is used to determine genotype-specific differences, we wondered to what extent epoxygenase and EH activities would be changed with varying AA concentrations. Particularly, we wanted to know how mEH and sEH activities adapt to varying degrees of epoxygenase activity. Since MS analysis revealed already high amounts of free AA levels in all liver homogenate samples, we switched to liver microsomes for these experiments, as these would allow for a better manipulation of experimental parameters. sEH KO and mEH KO liver microsomes were chosen, assuming that they contain exclusively one type of EH. This was tested in turnover assays with 11,12-EET as substrate in absence and presence of the selective sEH inhibitor tAUCB (mEH KO microsomes) and the selective mEH inhibitors TCPO/NSPA (sEH KO microsomes). tAUCB reduced diol formation in mEH KO microsomes by 98% (Fig. 4c), TCPO/NSPA in sEH KO microsomes by 97% (Fig. 4d), respectively, confirming that indeed exclusively sEH or mEH constitute the remaining EHs in these microsomes.

In a first AA experiment, KO liver microsomes were incubated with different concentrations of AA (1, 10, 30 μM) for 10, 30, and 60 min in presence of a regenerating system (isocitrate/isocitrate dehydrogenase) and 2 mM NADPH to guarantee permissive conditions for epoxygenases. The sum of generated [EETs and DHETs] was assumed to reflect epoxygenase activity, which increased steadily over time and with increasing AA concentrations. Epoxygenase activity was similar for mEH and sEH KO microsomes and neither genotype showed any indication for epoxygenase saturation, as concentration curves were linear with a constant slope (Fig. 4a, b). For the next experiments, incubation time was set to 10 min and mEH and sEH KO microsomes were again incubated with varying AA concentrations. With regard to DHET formation, sEH and mEH KO microsomes showed similar rates for 8,9- and 11,12-DHET, when lower AA concentrations (1 and 10 μM) were provided. But with increasing AA concentrations, the difference between the two genotypes increased drastically (Fig. 4e–g) until the DHET profile looked similar to the one with liver homogenates and 50 μM AA, as shown in Fig. 4a. Finally, in an alternative attempt to curb epoxygenase activity, we incubated the microsomes with 30 μM AA, but omitted the regenerating system including NADPH (Fig. 4h). Under these conditions, DHET formation rate was approx. 40 times lower than with regenerating system plus NADPH and mEH KO and sEH KO generated similar amounts of diols, indicating that mEH and sEH are equally efficient in hydrolyzing epoxides under these conditions.

### mEH and sEH distribution in mouse liver

**Fig. 5:**
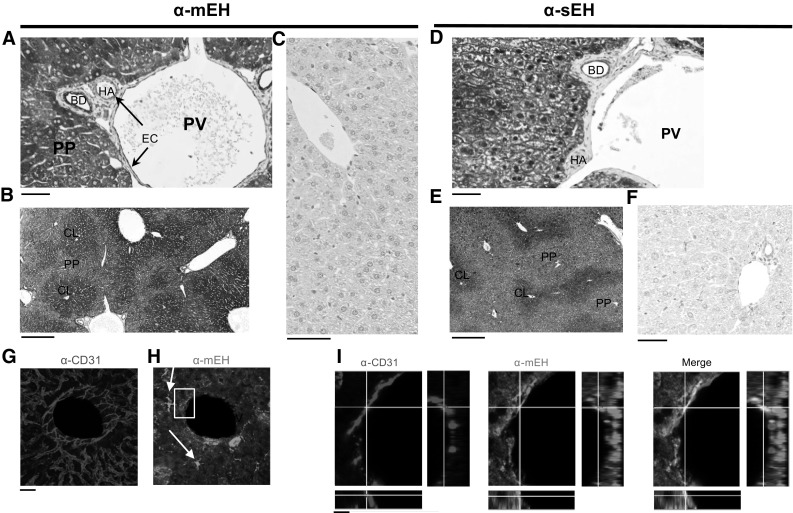
mEH and sEH expression in C57BL/6 mouse liver **a** mEH-positive cells include endothelial cells (EC), lining branches of the portal vein (PV) and hepatic artery (HA) (*arrows*), bile duct (BD) epithelial cells, and hepatocytes. **b** Centrilobular (CL) mEH expression in hepatocytes is slightly stronger than periportal (PP) expression. **c** No mEH expression is detected in the liver of mEH KO mice. **d** sEH expression is confined to hepatocytes and bile duct epithelium. **e** Similar to mEH, sEH shows a gradient of expression ranging from intense in the centrilobular areas to moderate in the periportal hepatocytes. **f** No sEH expression is detected in the liver of sEH KO mice. **g** Liver section showing a branch of the central vein stained for the endothelial cell marker CD31 and **h** mEH; note the mEH-positive Kupffer cells (*arrows*), which show stronger mEH immunoreactivity compared to the surrounding hepatocytes. **i** Detail from H (*box*). Endothelial cells in the crossline are positive for CD31 (*red*) and mEH (*green, middle panel*); colocalization shown in *yellow* (*right panel*). *Scale bar* in **a**, **c**, **d**, and **f** equals 50 μm. *Scale bar* in **b** and **e** equals 250 μm, in **g** and **h** 30 μm and in **i** 10 μm

To investigate in detail the distribution pattern of mEH and sEH in the mouse liver, IHC was applied to formalin-fixed paraffin-embedded liver sections obtained from both genders from C57BL/6 WT control, mEH KO, and sEH KO animals. In accordance with earlier studies (Kawabata et al. 1981), we found evidence of zonal distribution for both mEH and sEH with higher expression in hepatocytes in the perivenous and weaker expression in the periportal region. Overall, mEH had a more homogenous and widespread distribution compared to sEH. In addition to hepatocytes, mEH was also expressed in endothelial cells lining the branches of the hepatic artery and portal vein, Kupffer cells, and bile duct and gall bladder epithelial cells (Fig. 5a, b, g–i). sEH expression was confined to hepatocytes and epithelial cells of bile ducts and gall bladder (Fig. 5d, e). Distribution patterns for both mEH and sEH were similar in male and female mice. Furthermore, there was no difference in the sEH distribution pattern between WT and mEH KO livers, nor in the mEH distribution pattern between WT and sEH KO livers. Immunohistochemical stainings for mEH and sEH on livers of the correspondent KO mice showed no unspecific staining, confirming the specificity of the respective sEH and mEH antibody (Fig. 5c, f).

### Transcriptome analysis

**Fig. 6:**
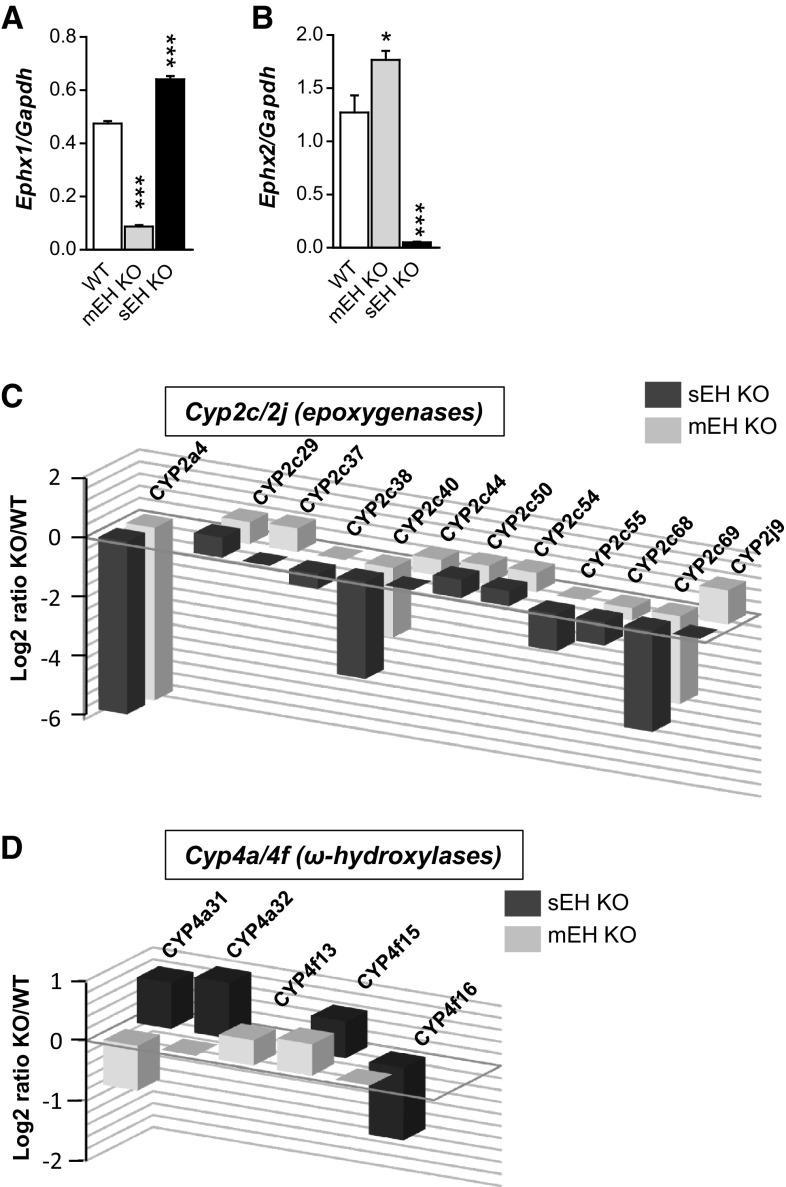
Changes in genes of the CYP-epoxygenase–EH pathway in mEH KO and sEH KO liver transcriptome. **a** *Ephx1*, encoding for the mEH protein, is 1.35-fold upregulated in sEH KO relative to WT liver. **b** *Ephx2*, encoding for the sEH protein, is 1.4-fold upregulated in mEH KO relative to WT liver. Note, that in WT liver *Ephx2* is 2.5 times more abundant than *Ephx1* (*n* = 3 for each genotype, 1-way ANOVA followed by Bonferroni post tests; only significant differences between WT and KO are indicated). **c** Expression changes in *Cyp2c* and *Cyp2j* genes, encoding for epoxygenases, are shown as Log2 ratio of the respective KO over WT; the C*yp* gene with the strongest change in expression (*Cyp2a4*) is displayed for comparison. A 1.5-fold increase relative to WT is represented by 0.5 on the Log2 ratio scale, e.g., see the *Cyp2c44* bar for mEH KO. Only *Cyp2c/2j* genes with statistically significant changes (cut-off *p* < 0.05) relative to WT are depicted as bar graphs. Note that for several of these genes, expression changes occur in parallel for both KO livers. **d** The same as **c** for ω-hydroxylase genes. Expression changes in the 20-HETE synthases only occur with low-expression ω-hydroxylase genes

Finally, the complete liver transcriptomes from C57BL/6 WT control, sEH KO, and mEH KO were compared to gain further insight on transcriptional effects caused by the deletion of EH gene transcripts (see Suppl. Table 3 and 4 for a list of the 50 most dysregulated genes in mEH KO and sEH KO livers). In WT liver, the gene expression ratio for *Ephx1:Ephx2* (with *Ephx1* encoding for mEH protein and *Ephx2* for sEH protein, respectively) was 1:2.6 (left columns in Fig. 6a, b). In mEH KO liver, gene expression of *Ephx2* was 1.4-fold higher relative to WT liver (Fig. 6b), indicating that transcriptional upregulation probably underlies the higher sEH protein expression and activity observed in mEH KO liver. Surprisingly, gene expression of *Ephx1* was also enhanced by factor 1.35 in sEH KO liver relative to WT (Fig. 6a). Genes encoding for other EHs, e.g., *Ephx3* and *Ephx4* were not present in any of the analyzed liver transcriptomes. Next, we focused on the changes in the expression levels of *Cyp* genes. We found the same 64 *Cyp* genes expressed in detectable amounts (statistically significant above background level) in WT, mEH KO, and sEH KO liver (Suppl. Table 5). *Cyp2e1* was by far the most abundant, irrespective of the genotype (Table 1). While most CYP proteins probably exert at least some epoxygenase activity (Kaspera and Totah 2009), members of the *Cyp2*c and *Cyp2j* subfamilies are commonly noted to exert the highest epoxygenase activity with AA. Which epoxygenase isoforms are particularly efficient in oxidizing linoleic acid, the precursor lipid for EpOMEs, is still unknown. Whereas the *Cyp2c* family was represented by 13 isoforms in C57BL/6 liver (*2c29*, *2c37*, *2c38*, *2c39*, *2c40*, *2c44*, *2c50*, *2c54*, *2c55*, *2c67*, *2c68*, *2c69*, *2c70*), only 3 *Cyp2j* isoenzymes were present (*2j5*, *2j6* and *2j9*). Overall, effects on *Cyp2c* and *Cyp2j* isoforms were disparate with certain isoforms either reduced, upregulated, or unchanged in KO relative to WT livers (Fig. 6c and Suppl. Table 5). However, the most abundant *Cyp* genes were dysregulated in parallel manner in both KO livers: *Cyp2c70* and *Cyp2j5* were unchanged, *Cyp2c29* and *Cyp2c50* significantly increased, and *Cyp2c68* decreased relative to WT (Fig. 6c, Table 1, and Suppl. Table 5). *Cyp4a* and *Cyp4f* isoforms code for ω-hydroxylases, which catalyze the formation of 20-HETE, a strong vasoconstrictor, and in many tissues the antagonist of EET-mediated actions. sEH deficiency mainly increased the expression of several isoenzymes (*4a31*, *4a32*, *4f13*, *4f15* with *p* < 0.05 as cut-off). Loss of mEH led to more inconsistent effects, causing upregulation of *Cyp4f13* and *Cyp4f15* and suppression of *Cyp4a31* (*p* < 0.05 as cut-off; Fig. 6d and Suppl. Table 5). Notably, neither mEH nor sEH deficiency affected the expression of the most abundant 20-HETE synthase genes such as *Cyp4a12a* and *Cyp4a12b* (see Suppl. Table 5 for details).

**Table 1:** Expression of *Cyp* genes in C57BL/6 liver in descending order, normalized to *Gapdh* gene expression

Isoenzyme	
*Cyp2e1*	13.53
*Cyp3a11*	5.81
*Cyp2f2*	2.85
*Cyp2d9*	2.76
*Cyp2c70*	2.72
*Cyp2c29*	2.60
*Cyp2c50*	2.02
*Cyp2j5*	1.77
*Cyp2a12*	1.54
*Cyp1a2*	1.51
*Cyp2d26*	1.41
*Cyp2c67*	1.18
*Cyp2d10*	1.16
*Cyp4v3*	1.14
*Cyp2a5*	1.10
*Cyp2c68*	1.07
*Cyp27a1*	1.00
*Cyp7b1*	0.92
*Cyp2c54*	0.87
*Cyp2c44*	0.86
*Cyp3a25*	0.80
*Cyp4f14*	0.77
*Cyp4a12a*	0.70
*Cyp2c37*	0.63
*Cyp51*	0.60
*Cyp8b1*	0.60
*Cyp2d22*	0.44
*Cyp3a13*	0.39
*Cyp2c40*	0.29
*Cyp7a1*	0.29
*Cyp2j6*	0.25
*Cyp4a12b*	0.22
*Cyp4f15*	0.216
*Cyp4a10*	0.195
*Cyp2a4*	0.179
*Cyp2c69*	0.150
*Cyp4f13*	0.122
*Cyp2d40*	0.111
*Cyp2c38*	0.086
*Cyp39a1*	0.067
*Cyp4a14*	0.063
*Cyp3a59*	0.050
*Cyp2r1*	0.043
*Cyp2u1*	0.037
*Cyp4a32*	0.037
*Cyp20a1*	0.031
*Cyp17a1*	0.026
*Cyp2c55*	0.023
*Cyp4b1*	0.022
*Cyp4f17*	0.015
*Cyp26b1*	0.014
*Cyp4a31*	0.013
*Cyp26a1*	0.009
*Cyp2j9*	0.008
*Cyp4f16*	0.006
*Cyp2c39*	0.006
*Cyp2a22*	0.004
*Cyp2g1*	0.004
*Cyp2b10*	0.003
*Cyp1a1*	0.003
*Cyp1b1*	0.002
*Cyp2s1*	0.002
*Cyp2d12*	0.002
*Cyp2b9*	0.001

## Discussion

Regarding the quantitative contribution to hepatic FA epoxide hydrolysis, the odds are clearly in favor of sEH: Based on the transcriptome data presented in this study, sEH expression is approximately 2.5 times higher than mEH expression in mouse liver and under saturating conditions, purified mouse sEH is between 24 times (8,9-EET) and 800 times (14,15-EET) faster in hydrolyzing the respective epoxides compared to mEH. Nevertheless, we found the plasma levels for two FA epoxides substantially decreased in mEH KO relative to WT animals, providing strong evidence for a substantial role of mEH in the metabolism of epoxy FAs in vivo. Moreover, the strong correlation between FA plasma levels and hepatic EH hydrolysis rates (Schuck et al. 2014) points to hepatic mEH as important player in FA hydrolysis.

Indeed, hepatic mEH exerts substantial FA epoxide hydrolase activity, as sEH KO livers still synthesize 65% (Fig. 4; Suppl. Table 2) of DHETs and DiHOMEs formed under the same conditions in WT livers. Several lines of evidence suggest that it is mEH that with high probability mediates the residual EH activity in sEH KO liver: (1) sEH deficiency in sEH KO mice was confirmed on protein level [sEH protein was no longer detectable (Luria et al. 2007)]; (2) genes of other known EHs were not expressed in C57BL/6 liver, excluding the possibility that EH3 or EH4 account for the hydrolysis; and, most importantly, (3) EH activity in sEH KO microsomes could almost completely be blocked by a combination of mEH blockers (Fig. 5d). There is a low residual probability, however, that one of the upregulated genes in sEH KO liver (Suppl. Table 4), whose protein product is unknown, encodes for a so far uncharacterized EH.

Interestingly, a recent report corroborated our results that sEH KO liver exhibits high EH activity. In the study by Zhu and colleagues (Zhu et al. 2016), hepatic levels of FA epoxides and diols were determined by subjecting WT and sEH KO livers to alkaline lysis; DHET and DiHOME levels were similar between WT and sEH KO with the exception of 14,15-DHET, which was significantly lower in sEH KO liver. We also found strong variations in mEH-mediated diol formation in sEH KO liver depending on the parental epoxides. The difference between the respective formation rates in WT and sEH KO was highest for 12,13-DiHOME, followed by 5,6-DHET and 14,15-DHET, which is in accord with the fact that the respective epoxides are the least preferred substrates of mEH. The difference in the formation rates between WT and sEH KO was comparatively small for 8,9- and 11,12-DHET as well as for 9,10-DiHOME, indicating that the parental epoxides constitute the best substrates for mEH in the present series of compounds. However, mEH KO plasma only showed reduced hydrolysis ratios for 8,9-DHET:EET and 9,10-DiHOME:EpOME. Thus, if both enzymes are present in liver, sEH is probably the predominant EH for the conversion of 5,6-, 11,12-, and 14,15-EET as well as 12,13-EpOME and mEH the predominant EH for hydrolyzing 8,9-EET and 9,10-EpOME.

The preference profile for sEH indicates a low catalytic efficiency (reflecting the enzyme’s preference) for 8,9-EET, but a particularly high one for 9,10-EpOME (Morisseau et al. 2010). In hepatocytes, where both EH are present, sEH should hence dominate the hydrolysis of 9,10-EpOME due to its higher activity with this substrate. Despite the strong relationship between hepatic EH activity and FA hydrolysis rates in plasma, it may be that plasma 9,10-DiHOME is formed in other cells than hepatocytes. These cells might possibly only express mEH. Classically, DiHOMEs are formed during neutrophilic outburst (Hayakawa et al. 1986) and by adipocytes (Lynes et al. 2017). mEH expression seems principally to be more widespread compared to the one of sEH, comprising not necessarily more tissues, but cell types. Notably, mEH is found in some immunoreactive cells such as the liver-resident macrophages (Kupffer cells) and in brain in activated microglia (unpublished data) as well as in endothelial cells of many tissues (Marowsky et al. 2009)—with none of these cell types expressing sEH to our knowledge. However, if any of these mEH-expressing cells synthesize DiHOMEs remains to be elucidated.

The transcriptome data provide further proof for a critical role of mEH in endogenous lipid metabolism. Loss of either EH induced the transcriptional upregulation of the residual EH gene to a similar degree (1.3- and 1.4-fold, respectively), which is indicative of the notion that they share the same substrates. However, with regard to xenobiotic substrates, mEH and sEH display complementarity: Only sEH can hydrolyze trans-substituted compounds, but is restricted to “slim” substrates, while mEH is capable of hydrolyzing bulky hydrophobic substrates as long as they are not trans-substituted. This suggests that their shared substrates are rather of endogenous origin, e.g., FA epoxides.

Moreover, the principal CYPs and CYP epoxygenases were dysregulated in parallel manner in both KO transcriptomes. The by far most abundant *Cyp* in C57BL/6 WT liver (*Cyp2e1*) was increased in mEH KO (1.8-fold) and sEH KO (1.6-fold) relative to WT (Suppl. Table 5). According to our transcriptome analysis, *Cyp2c70*, *Cyp2c29*, *Cyp2c50*, *Cyp2j5*, and *Cyp2c68* constitute the five most abundant epoxygenases in C57BL/6 WT liver, a result largely in line with previous studies (Schuck et al. 2014; Theken et al. 2011). The expression of these five key epoxygenases was altered in identical manner in both KO mice. However, since gene expression changes occurred in both directions, it was impossible to deduce if EH loss leads as net effect to less or more hepatic epoxygenase activity. The biochemical assays yielded conflicting results: In assays with mEH and sEH KO microsomes, the sum of generated [EETs and DHETs] was similar between genotypes, suggesting that the combined capacity of epoxygenases and EHs was unaltered between the two KO strains. However, in experiments with liver tissue, mEH KO liver formed substantially more diols (epoxides were not detectable), whereas sEH KO liver formed less [epoxides and diols] compared to WT. It should be noted that sEH levels differ between these two preparations (the content of the cytosol-residing sEH is reduced in microsomal fractions, but not in liver homogenates) and that hence sEH or diol levels may influence the availability of AA and epoxygenase activity.

The vasoconstrictor 20-HETE is generated by Cyp4a and Cyp4f isoforms (also termed ω-hydroxylases) and commonly considered to play an opposing role to the vasodilatory EETs. sEH deficiency leads to transcriptional upregulation of 20-HETE generating CYPs exclusively in kidney, but not in liver (Luria et al. 2007; Zhu et al. 2016). We found that the most abundantly expressed ω-hydroxylases with reportedly high catalytic efficiency with AA [*Cyp4a12a*, *Cyp4a12b* (Muller et al. 2007), *Cyp4f15 *(Schuck et al. 2014)] are either not affected by loss of mEH or sEH or upregulated in both KO livers (*Cyp4f15*) compared to WT. The AA capacity assay further revealed that the hepatic formation rate for 20-HETE was similar across genotypes, which is consistent with earlier studies (Luria et al. 2007; Zhu et al. 2016). Interestingly, in liver and lung, EETs were reported to exert vasoconstricting rather than vasodilatory effects, thus not counteracting 20-HETE action but rather adding to it (Sacerdoti et al. 2015). This might be a possible reason why the generation of 20-HETE is unchanged in sEH KO relative to WT liver, but increased in sEH KO kidney.

Our data also indicate that certain conditions modulate the contribution of mEH to FA epoxide hydrolysis. Notably, low epoxide levels favor mEH-mediated over sEH-mediated hydrolysis. This is possibly due to the lower *K*_M_ values (translating into higher affinity) displayed by mEH with all EETs compared to sEH (Decker et al. 2012). mEH-mediated hydrolysis may profit from one additional fact: The enzyme is localized in close physical proximity to expoxygenases to mEH in intact cells, allowing probably for the direct transfer of the substrate from epoxygenases in a process called substrate channeling (Orjuela et al. 2017). This effect, however, only comes into full play when the cell compartmentalization is left intact (e.g., with untreated cells and in vivo). In liver homogenates and microsomes, where cell compartmentalization is largely destroyed, epoxides may diffuse more easily and thus increase the probability for being subjected to sEH-mediated hydrolysis. This may explain why mEH effects are mainly seen in vivo (Marowsky et al. 2016), but not in capacity assays with homogenates or cellular fraction with excess AA (Luria et al. 2007). Furthermore, the observed mEH activity with FA epoxides might explain recent findings that human mEH polymorphisms correlate with the susceptibility to develop preeclampsia (Zusterzeel et al. 2001).

Taken together, our data clearly support a significant role for mEH in lipid signaling, particularly in the hydrolysis of 8,9-EET and 9,10-EpOME. Yet it should be emphasized that mEH-mediated hydrolysis does not extend to endocannabinoids, as suggested recently (Nithipatikom et al. 2014), because the reported hydrolytic activity with 2-Arachidonoylglycerol was not attributable to mEH (Arand and Marowsky 2016). Further studies are warranted to define the exact role of mEH in endogenous FA hydrolysis and in the regulation of physiological processes related to this.

## Electronic supplementary material

Below is the link to the electronic supplementary material.

Supplementary material 1 (PPTX 71 kb)

Supplementary material 2 (DOCX 22 kb)

Supplementary material 3 (DOCX 23 kb)

Supplementary material 4 (PPTX 73 kb)

Supplementary material 5 (PPTX 70 kb)

## Abbreviations

AA: Arachidonic acid
CL: Centrilobular
CYP: Cytochrome P450
EC: Endothelial cell
EET: Epoxyeicosatrienoic acid
EH: Epoxide hydrolase
EpOME: Epoxy-octadecenoic acid
FA: Fatty acid
DiHOME: Dihydroxy-octadecenoic acid
DHET: Dihydroxy-eicosatrienoic acid
DMBA: 7,12-Dimethylbenz[a]anthracene
fdr: False discovery rate
HETE: Hydroxyeicosatetraenoic acid
mEH: Microsomal epoxide hydrolase
NSPA: 2-Nonylsulfanyl-propanamide
PP: Periportal
PV: Portal vein
sEH: Soluble epoxide hydrolase
tAUCB: Trans-4-[4-(3-adamantan-1-y1-ureido)-cyclohexyloxy]-benzoic acid
TCPO: 1,1,1-Trichloropropene oxide
