# Supplementary material for: Beyond detoxification: a role for mouse mEH in the hepatic metabolism of endogenous lipids
Source: Arch Toxicol. 2017 Oct 3;91(11):3571–85. doi: 10.1007/s00204-017-2060-4 (PMC5696502; doi:10.1007/s00204-017-2060-4)
Supplement: Supplementary file 1 — Supplementary material 1 (PPTX 71 kb) [file 204_2017_2060_MOESM1_ESM.pptx]

## Slide 1
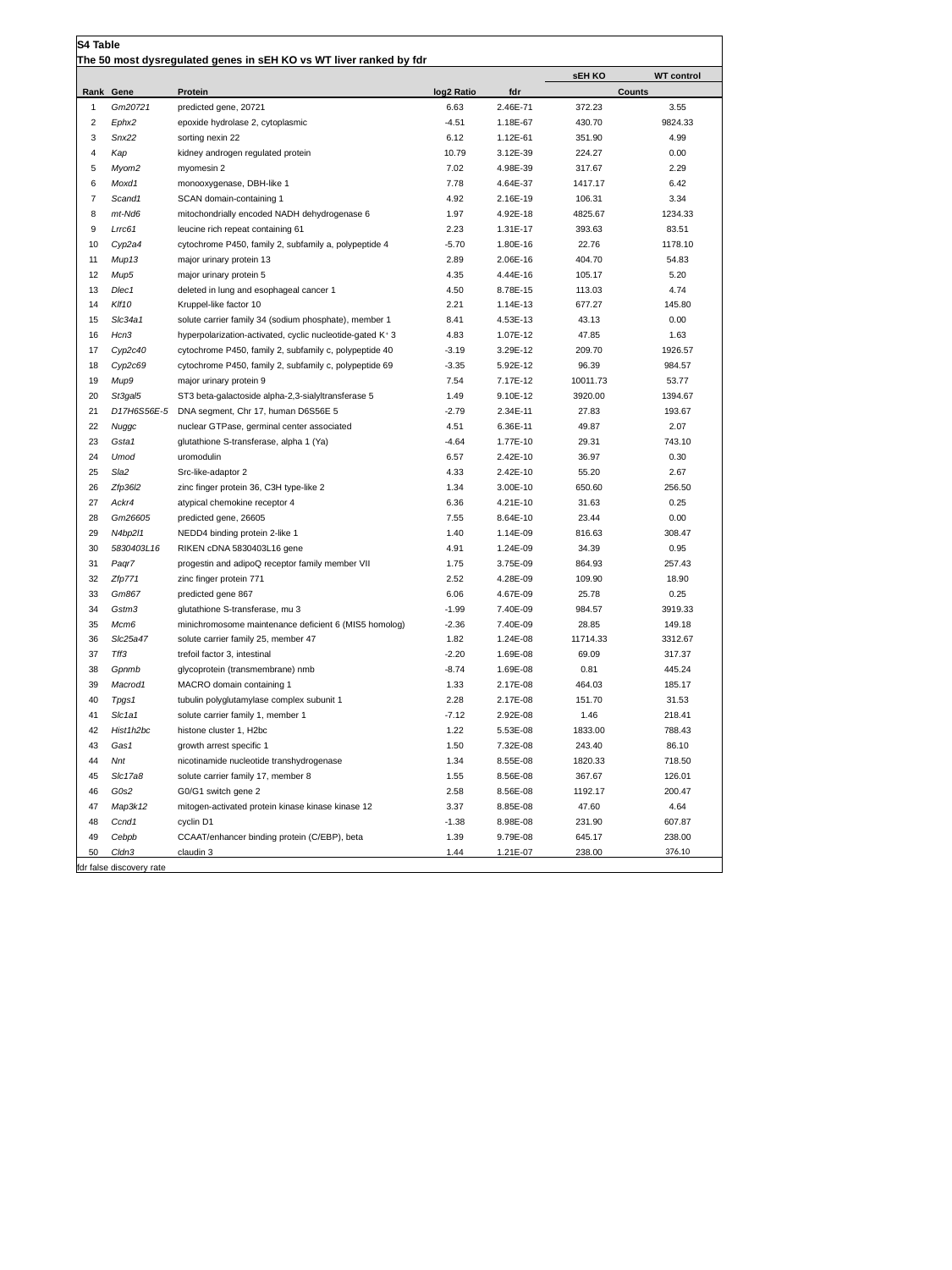

| S4 Table | | | | | | |
| --- | --- | --- | --- | --- | --- | --- |
| The 50 most dysregulated genes in sEH KO vs WT liver ranked by fdr | | | | | | |
| | | | | | sEH KO | WT control |
| Rank | Gene | Protein | log2 Ratio | fdr | Counts | |
| 1 | Gm20721 | predicted gene, 20721 | 6.63 | 2.46E-71 | 372.23 | 3.55 |
| 2 | Ephx2 | epoxide hydrolase 2, cytoplasmic | -4.51 | 1.18E-67 | 430.70 | 9824.33 |
| 3 | Snx22 | sorting nexin 22 | 6.12 | 1.12E-61 | 351.90 | 4.99 |
| 4 | Kap | kidney androgen regulated protein | 10.79 | 3.12E-39 | 224.27 | 0.00 |
| 5 | Myom2 | myomesin 2 | 7.02 | 4.98E-39 | 317.67 | 2.29 |
| 6 | Moxd1 | monooxygenase, DBH-like 1 | 7.78 | 4.64E-37 | 1417.17 | 6.42 |
| 7 | Scand1 | SCAN domain-containing 1 | 4.92 | 2.16E-19 | 106.31 | 3.34 |
| 8 | mt-Nd6 | mitochondrially encoded NADH dehydrogenase 6 | 1.97 | 4.92E-18 | 4825.67 | 1234.33 |
| 9 | Lrrc61 | leucine rich repeat containing 61 | 2.23 | 1.31E-17 | 393.63 | 83.51 |
| 10 | Cyp2a4 | cytochrome P450, family 2, subfamily a, polypeptide 4 | -5.70 | 1.80E-16 | 22.76 | 1178.10 |
| 11 | Mup13 | major urinary protein 13 | 2.89 | 2.06E-16 | 404.70 | 54.83 |
| 12 | Mup5 | major urinary protein 5 | 4.35 | 4.44E-16 | 105.17 | 5.20 |
| 13 | Dlec1 | deleted in lung and esophageal cancer 1 | 4.50 | 8.78E-15 | 113.03 | 4.74 |
| 14 | Klf10 | Kruppel-like factor 10 | 2.21 | 1.14E-13 | 677.27 | 145.80 |
| 15 | Slc34a1 | solute carrier family 34 (sodium phosphate), member 1 | 8.41 | 4.53E-13 | 43.13 | 0.00 |
| 16 | Hcn3 | hyperpolarization-activated, cyclic nucleotide-gated K+ 3 | 4.83 | 1.07E-12 | 47.85 | 1.63 |
| 17 | Cyp2c40 | cytochrome P450, family 2, subfamily c, polypeptide 40 | -3.19 | 3.29E-12 | 209.70 | 1926.57 |
| 18 | Cyp2c69 | cytochrome P450, family 2, subfamily c, polypeptide 69 | -3.35 | 5.92E-12 | 96.39 | 984.57 |
| 19 | Mup9 | major urinary protein 9 | 7.54 | 7.17E-12 | 10011.73 | 53.77 |
| 20 | St3gal5 | ST3 beta-galactoside alpha-2,3-sialyltransferase 5 | 1.49 | 9.10E-12 | 3920.00 | 1394.67 |
| 21 | D17H6S56E-5 | DNA segment, Chr 17, human D6S56E 5 | -2.79 | 2.34E-11 | 27.83 | 193.67 |
| 22 | Nuggc | nuclear GTPase, germinal center associated | 4.51 | 6.36E-11 | 49.87 | 2.07 |
| 23 | Gsta1 | glutathione S-transferase, alpha 1 (Ya) | -4.64 | 1.77E-10 | 29.31 | 743.10 |
| 24 | Umod | uromodulin | 6.57 | 2.42E-10 | 36.97 | 0.30 |
| 25 | Sla2 | Src-like-adaptor 2 | 4.33 | 2.42E-10 | 55.20 | 2.67 |
| 26 | Zfp36l2 | zinc finger protein 36, C3H type-like 2 | 1.34 | 3.00E-10 | 650.60 | 256.50 |
| 27 | Ackr4 | atypical chemokine receptor 4 | 6.36 | 4.21E-10 | 31.63 | 0.25 |
| 28 | Gm26605 | predicted gene, 26605 | 7.55 | 8.64E-10 | 23.44 | 0.00 |
| 29 | N4bp2l1 | NEDD4 binding protein 2-like 1 | 1.40 | 1.14E-09 | 816.63 | 308.47 |
| 30 | 5830403L16 | RIKEN cDNA 5830403L16 gene | 4.91 | 1.24E-09 | 34.39 | 0.95 |
| 31 | Paqr7 | progestin and adipoQ receptor family member VII | 1.75 | 3.75E-09 | 864.93 | 257.43 |
| 32 | Zfp771 | zinc finger protein 771 | 2.52 | 4.28E-09 | 109.90 | 18.90 |
| 33 | Gm867 | predicted gene 867 | 6.06 | 4.67E-09 | 25.78 | 0.25 |
| 34 | Gstm3 | glutathione S-transferase, mu 3 | -1.99 | 7.40E-09 | 984.57 | 3919.33 |
| 35 | Mcm6 | minichromosome maintenance deficient 6 (MIS5 homolog) | -2.36 | 7.40E-09 | 28.85 | 149.18 |
| 36 | Slc25a47 | solute carrier family 25, member 47 | 1.82 | 1.24E-08 | 11714.33 | 3312.67 |
| 37 | Tff3 | trefoil factor 3, intestinal | -2.20 | 1.69E-08 | 69.09 | 317.37 |
| 38 | Gpnmb | glycoprotein (transmembrane) nmb | -8.74 | 1.69E-08 | 0.81 | 445.24 |
| 39 | Macrod1 | MACRO domain containing 1 | 1.33 | 2.17E-08 | 464.03 | 185.17 |
| 40 | Tpgs1 | tubulin polyglutamylase complex subunit 1 | 2.28 | 2.17E-08 | 151.70 | 31.53 |
| 41 | Slc1a1 | solute carrier family 1, member 1 | -7.12 | 2.92E-08 | 1.46 | 218.41 |
| 42 | Hist1h2bc | histone cluster 1, H2bc | 1.22 | 5.53E-08 | 1833.00 | 788.43 |
| 43 | Gas1 | growth arrest specific 1 | 1.50 | 7.32E-08 | 243.40 | 86.10 |
| 44 | Nnt | nicotinamide nucleotide transhydrogenase | 1.34 | 8.55E-08 | 1820.33 | 718.50 |
| 45 | Slc17a8 | solute carrier family 17, member 8 | 1.55 | 8.56E-08 | 367.67 | 126.01 |
| 46 | G0s2 | G0/G1 switch gene 2 | 2.58 | 8.56E-08 | 1192.17 | 200.47 |
| 47 | Map3k12 | mitogen-activated protein kinase kinase kinase 12 | 3.37 | 8.85E-08 | 47.60 | 4.64 |
| 48 | Ccnd1 | cyclin D1 | -1.38 | 8.98E-08 | 231.90 | 607.87 |
| 49 | Cebpb | CCAAT/enhancer binding protein (C/EBP), beta | 1.39 | 9.79E-08 | 645.17 | 238.00 |
| 50 | Cldn3 | claudin 3 | 1.44 | 1.21E-07 | 238.00 | 376.10 |
| fdr false discovery rate | | | | | | |
