# Supplementary material for: Beyond detoxification: a role for mouse mEH in the hepatic metabolism of endogenous lipids
Source: Arch Toxicol. 2017 Oct 3;91(11):3571–85. doi: 10.1007/s00204-017-2060-4 (PMC5696502; doi:10.1007/s00204-017-2060-4)
Supplement: Supplementary file 2 — Supplementary material 2 (DOCX 22 kb) [file 204_2017_2060_MOESM2_ESM.docx]

| **S1 Table** |  |  |  |  |  |  |  |  |  |  |  |  |  |
| --- | --- | --- | --- | --- | --- | --- | --- | --- | --- | --- | --- | --- | --- |
| **Levels of selected oxylipins in plasma from C57BL/6 WT, mEH KO and sEH KO mice** | | | | | | | | | | | |  |  |
|  |  |  |  |  |  |  |  |  |  |  |  |  |  |
|  |  |  |  |  |  |  |  |  |  |  |  |  |  |
| (pmol/ml ) |  | **WT** | | |  | **mEH KO** | | |  | **sEH KO** | | |  |
| *Cyp epoxygenase* | |  |  |  |  |  |  |  |  |  |  |  |  |
| *metabolites of arachidonic acid* | |  |  |  |  |  |  |  |  |  |  |  |  |
| 5,6 EET |  | 0.78 | ± | 0.18 |  | 0.58 | ± | 0.22 |  | 0.86 | ± | 0.09 |  |
| 8,9 EET |  | 2.15 | ± | 0.39 |  | 2.37 | ± | 0.67 |  | 2.93 | ± | 0.30 |  |
| 11,12 EET |  | 6.93 | ± | 0.70 |  | 8.05 | ± | 2.16 |  | 6.06 | ± | 0.81 |  |
| 14,15 EET |  | 3.68 | ± | 0.19 |  | 2.88 | ± | 0.45 |  | 7.05 | ± | 0.47*** |  |
|  |  |  |  |  |  |  |  |  |  |  |  |  |  |
| *Cyp epoxygenase* | |  |  |  |  |  |  |  |  |  |  |  |  |
| *metabolites of linoleic acid* | |  |  |  |  |  |  |  |  |  |  |  |  |
| 9,10 EpoME |  | 9.92 | ± | 2.00 |  | 12.13 | ± | 5.61 |  | 62.20 | ± | 7.20*** |  |
| 12,13 EpoME | | 12.10 | ± | 1.02 |  | 9.96 | ± | 1.20 |  | 111.50 | ± | 10.50*** |  |
|  |  |  |  |  |  |  |  |  |  |  |  |  |  |
| *Epoxide hydrolase* | |  |  |  |  |  |  |  |  |  |  |  |  |
| *metabolites* | |  |  |  |  |  |  |  |  |  |  |  |  |
| (5,6 DHET) |  | 0.88 | ± | 0.07 |  | 1.02 | ± | 0.14 |  | 0.42 | ± | 0.07*** |  |
| 8,9 DHET |  | 1.24 | ± | 0.20 |  | 0.52 | ± | 0.01** |  | 0.71 | ± | 0.12* |  |
| 11,12 DHET |  | 3.06 | ± | 0.38 |  | 3.26 | ± | 0.28 |  | 2.55 | ± | 0.35 |  |
| 14,15 DHET |  | 2.18 | ± | 0.27 |  | 2.33 | ± | 0.13 |  | 0.88 | ± | 0.12*** |  |
| 9,10 DiHOME | | 45.63 | ± | 2.80 |  | 25.76 | ± | 1.76** |  | 47.10 | ± | 6.26 |  |
| 12,13 DiHOME | | 27.96 | ± | 2.05 |  | 30.78 | ± | 2.51 |  | 6.94 | ± | 1.40*** |  |
|  |  |  |  |  |  |  |  |  |  |  |  |  |  |
| ω-*hydroxylase* | |  |  |  |  |  |  |  |  |  |  |  |  |
| *metabolites* | |  |  |  |  |  |  |  |  |  |  |  |  |
| 20-HETE |  | 1.84 | ± | 0.32 |  | 1.92 | ± | 0.22 |  | 2.10 | ± | 0.48 |  |
|  |  |  |  |  |  |  |  |  |  |  |  |  |  |
| *Lipoxygenase metabolites* | |  |  |  |  |  |  |  |  |  |  |  |  |
| *of arachidonic acid* | |  |  |  |  |  |  |  |  |  |  |  |  |
| 5-HETE |  | 4.27 | ± | 0.56 |  | 4.26 | ± | 1.89 |  | 3.24 | ± | 0.33 |  |
| 8-HETE |  | 2.04 | ± | 0.23 |  | 2.03 | ± | 0.60 |  | 1.89 | ± | 0.15 |  |
| 12-HETE |  | 41.17 | ± | 10.40 |  | 48.05 | ± | 22.41 |  | 48.93 | ± | 11.42 |  |
| 15-HETE |  | 2.93 | ± | 0.71 |  | 2.22 | ± | 0.33 |  | 2.20 | ± | 0.19 |  |
|  |  |  |  |  |  |  |  |  |  |  |  |  |  |
| Mean values ± SEM are given. *n* equals 5 for all genotypes and metabolites. | | | | | | | | | | | |  |  |
| Mean values of mEH KO and sEH KO metabolites were compared to the respective WT metabolite. | | | | | | | | | | | | | |
| Asterisks indicate the significant differences obtained from these comparisons, using a | | | | | | | | | | | | | |
| 1-way ANOWA followed by Dunnett's Multiple Comparison test. p<0.05*, p<0.01**, p<0.001***. | | | | | | | | | | | | | |
