# Supplementary material for: Beyond detoxification: a role for mouse mEH in the hepatic metabolism of endogenous lipids
Source: Arch Toxicol. 2017 Oct 3;91(11):3571–85. doi: 10.1007/s00204-017-2060-4 (PMC5696502; doi:10.1007/s00204-017-2060-4)
Supplement: Supplementary file 3 — Supplementary material 3 (DOCX 23 kb) [file 204_2017_2060_MOESM3_ESM.docx]

| **S2 Table** |  |  |  |  |  |  |  |  |  |  |  |  |  |
| --- | --- | --- | --- | --- | --- | --- | --- | --- | --- | --- | --- | --- | --- |
| **Formation rate for selected oxylipins in liver from WT, mEH KO and sEH KO mice** | | | | | | | | | | | |  |  |
|  |  |  |  |  |  |  |  |  |  |  |  |  |  |
|  |  |  |  |  |  |  |  |  |  |  |  |  |  |
| (pmol/  mg organ*30 min) |  | **WT** | | |  | **mEH KO** | | |  | **sEH KO** | | |  |
| *CYP epoxygenase* | |  |  |  |  |  |  |  |  |  |  |  |  |
| *metabolites of arachidonic acid* | |  |  |  |  |  |  |  |  |  |  |  |  |
| 5,6 EET |  | #) |  |  |  | #) |  |  |  | #) |  |  |  |
| 8,9 EET |  | #) |  |  |  | #) |  |  |  | 0.27 | ± | 0.03(***) |  |
| 11,12 EET |  | #) |  |  |  | #) |  |  |  | 0.63 | ± | 0.08(***) |  |
| 14,15 EET |  | #) |  |  |  | #) |  |  |  | 1.82 | ± | 0.16(***) |  |
|  |  |  |  |  |  |  |  |  |  |  |  |  |  |
| *CYP epoxygenase* | |  |  |  |  |  |  |  |  |  |  |  |  |
| *metabolites of linoleic acid* | |  |  |  |  |  |  |  |  |  |  |  |  |
| 9,10 EpOME |  | #) |  |  |  | #) |  |  |  | 0.59 | ± | 0.11(***) |  |
| 12,13 EpOME | | #) |  |  |  | #) |  |  |  | 2.15 | ± | 0.23(***) |  |
|  |  |  |  |  |  |  |  |  |  |  |  |  |  |
| *Epoxide hydrolase* | |  |  |  |  |  |  |  |  |  |  |  |  |
| *metabolites* | |  |  |  |  |  |  |  |  |  |  |  |  |
| (5,6 DHET) |  | 2.56 | ± | 0.23 |  | 2.86 | ± | 0.27 |  | 1.36 | ± | 0.09* |  |
| 8,9 DHET |  | 4.12 | ± | 0.39 |  | 5.05 | ± | 0.27 |  | 2.52 | ± | 0.10* |  |
| 11,12 DHET |  | 7.12 | ± | 0.51 |  | 8.45 | ± | 0.26** |  | 5.79 | ± | 0.15* |  |
| 14,15 DHET |  | 5.01 | ± | 0.25 |  | 5.60 | ± | 0.29 |  | 2.14 | ± | 0.15*** |  |
| 9,10 DiHOME | | 3.10 | ± | 0.30 |  | 4.45 | ± | 0.16* |  | 4.10 | ± | 0.36 |  |
| 12,13 DiHOME | | 2.10 | ± | 0.05 |  | 2.63 | ± | 0.03* |  | 0.28 | ± | 0.02*** |  |
|  |  |  |  |  |  |  |  |  |  |  |  |  |  |
| ω-*hydroxylase* | |  |  |  |  |  |  |  |  |  |  |  |  |
| *metabolites* | |  |  |  |  |  |  |  |  |  |  |  |  |
| 20-HETE |  | 0.45 | ± | 0.03 |  | 0.40 | ± | 0.04 |  | 0.53 | ± | 0.04 |  |
|  |  |  |  |  |  |  |  |  |  |  |  |  |  |
| *Lipoxygenase metabolites* | |  |  |  |  |  |  |  |  |  |  |  |  |
| *of arachidonic acid* | |  |  |  |  |  |  |  |  |  |  |  |  |
| 5-HETE |  | 0.19 | ± | 0.04 |  | 0.17 | ± | 0.04 |  | 0.07 | ± | 0.02 |  |
| 8-HETE |  | 0.72 | ± | 0.06 |  | 0.97 | ± | 0.14 |  | 0.74 | ± | 0.18 |  |
| 12-HETE |  | #) |  |  |  | #) |  |  |  | #) |  |  |  |
| 15-HETE |  | #) |  |  |  | #) |  |  |  | #) |  |  |  |
|  |  |  |  |  |  |  |  |  |  |  |  |  |  |
| #) After subtraction of the background level of the respective metabolite from control samples (incubated in absence of AA), either negative values or values below the limit of quantification (0.005 pmol/mg organ) were obtained.  Mean values ± SEM are given. *n* equals 5 for all genotypes and metabolites. | | | | | | | | | | | |  |  |
| Mean values of mEH KO and sEH KO metabolites were compared to the respective WT metabolite. | | | | | | | | | | | | | |
| Asterisks indicate the significant differences obtained from these comparisons, using a | | | | | | | | | | | | | |
| 1-way ANOWA followed by Dunnett's Multiple Comparison test. p<0.05*, p<0.01**, <0.001***. | | | | | | | | | | | | | |
