# Supplementary material for: Beyond detoxification: a role for mouse mEH in the hepatic metabolism of endogenous lipids
Source: Arch Toxicol. 2017 Oct 3;91(11):3571–85. doi: 10.1007/s00204-017-2060-4 (PMC5696502; doi:10.1007/s00204-017-2060-4)
Supplement: Supplementary file 4 — Supplementary material 4 (PPTX 73 kb) [file 204_2017_2060_MOESM4_ESM.pptx]

## Slide 1
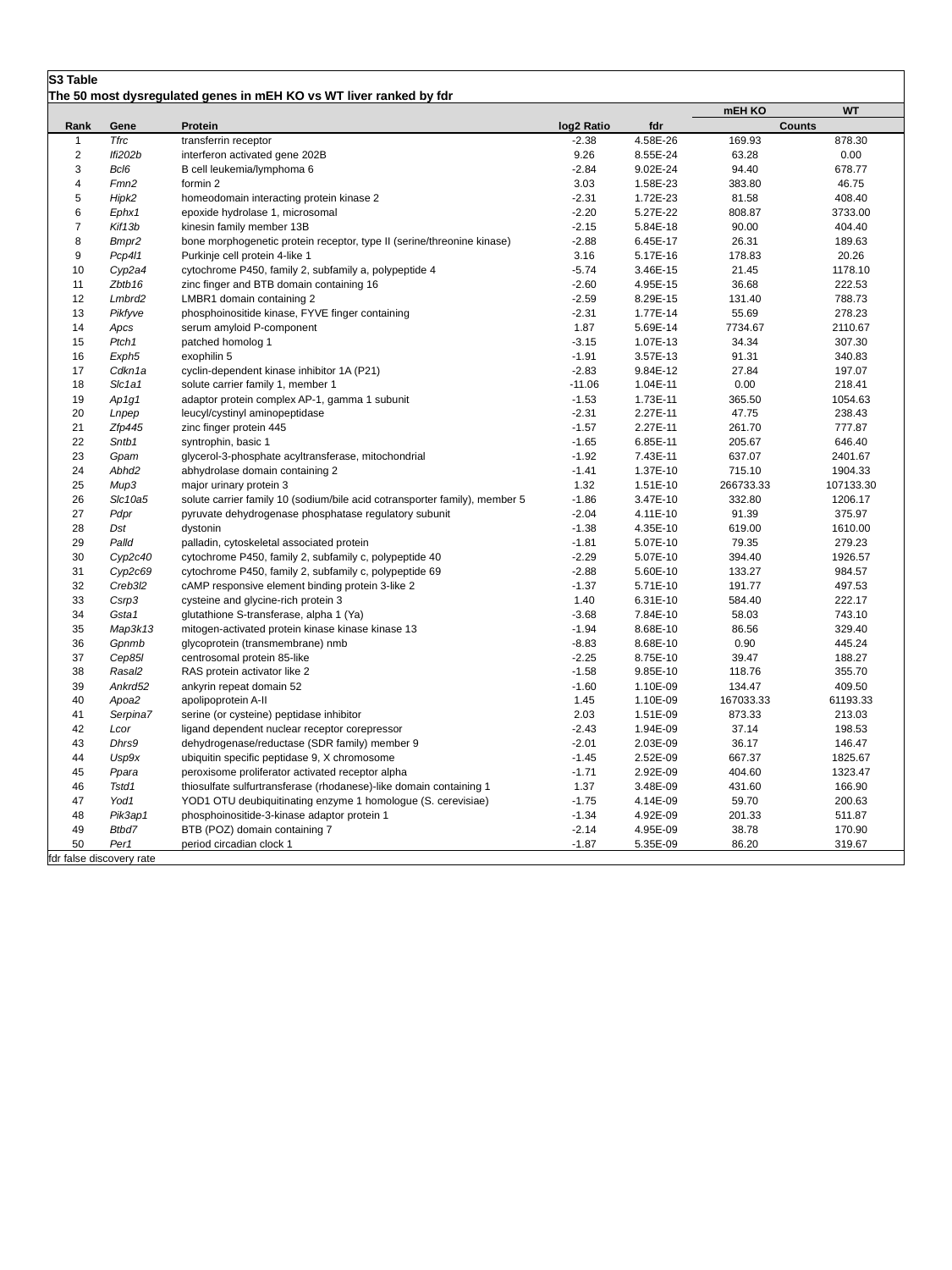

| S3 Table | | | | | | |
| --- | --- | --- | --- | --- | --- | --- |
| The 50 most dysregulated genes in mEH KO vs WT liver ranked by fdr | | | | | | |
| | | | | | mEH KO | WT |
| Rank | Gene | Protein | log2 Ratio | fdr | Counts | |
| 1 | Tfrc | transferrin receptor | -2.38 | 4.58E-26 | 169.93 | 878.30 |
| 2 | Ifi202b | interferon activated gene 202B | 9.26 | 8.55E-24 | 63.28 | 0.00 |
| 3 | Bcl6 | B cell leukemia/lymphoma 6 | -2.84 | 9.02E-24 | 94.40 | 678.77 |
| 4 | Fmn2 | formin 2 | 3.03 | 1.58E-23 | 383.80 | 46.75 |
| 5 | Hipk2 | homeodomain interacting protein kinase 2 | -2.31 | 1.72E-23 | 81.58 | 408.40 |
| 6 | Ephx1 | epoxide hydrolase 1, microsomal | -2.20 | 5.27E-22 | 808.87 | 3733.00 |
| 7 | Kif13b | kinesin family member 13B | -2.15 | 5.84E-18 | 90.00 | 404.40 |
| 8 | Bmpr2 | bone morphogenetic protein receptor, type II (serine/threonine kinase) | -2.88 | 6.45E-17 | 26.31 | 189.63 |
| 9 | Pcp4l1 | Purkinje cell protein 4-like 1 | 3.16 | 5.17E-16 | 178.83 | 20.26 |
| 10 | Cyp2a4 | cytochrome P450, family 2, subfamily a, polypeptide 4 | -5.74 | 3.46E-15 | 21.45 | 1178.10 |
| 11 | Zbtb16 | zinc finger and BTB domain containing 16 | -2.60 | 4.95E-15 | 36.68 | 222.53 |
| 12 | Lmbrd2 | LMBR1 domain containing 2 | -2.59 | 8.29E-15 | 131.40 | 788.73 |
| 13 | Pikfyve | phosphoinositide kinase, FYVE finger containing | -2.31 | 1.77E-14 | 55.69 | 278.23 |
| 14 | Apcs | serum amyloid P-component | 1.87 | 5.69E-14 | 7734.67 | 2110.67 |
| 15 | Ptch1 | patched homolog 1 | -3.15 | 1.07E-13 | 34.34 | 307.30 |
| 16 | Exph5 | exophilin 5 | -1.91 | 3.57E-13 | 91.31 | 340.83 |
| 17 | Cdkn1a | cyclin-dependent kinase inhibitor 1A (P21) | -2.83 | 9.84E-12 | 27.84 | 197.07 |
| 18 | Slc1a1 | solute carrier family 1, member 1 | -11.06 | 1.04E-11 | 0.00 | 218.41 |
| 19 | Ap1g1 | adaptor protein complex AP-1, gamma 1 subunit | -1.53 | 1.73E-11 | 365.50 | 1054.63 |
| 20 | Lnpep | leucyl/cystinyl aminopeptidase | -2.31 | 2.27E-11 | 47.75 | 238.43 |
| 21 | Zfp445 | zinc finger protein 445 | -1.57 | 2.27E-11 | 261.70 | 777.87 |
| 22 | Sntb1 | syntrophin, basic 1 | -1.65 | 6.85E-11 | 205.67 | 646.40 |
| 23 | Gpam | glycerol-3-phosphate acyltransferase, mitochondrial | -1.92 | 7.43E-11 | 637.07 | 2401.67 |
| 24 | Abhd2 | abhydrolase domain containing 2 | -1.41 | 1.37E-10 | 715.10 | 1904.33 |
| 25 | Mup3 | major urinary protein 3 | 1.32 | 1.51E-10 | 266733.33 | 107133.30 |
| 26 | Slc10a5 | solute carrier family 10 (sodium/bile acid cotransporter family), member 5 | -1.86 | 3.47E-10 | 332.80 | 1206.17 |
| 27 | Pdpr | pyruvate dehydrogenase phosphatase regulatory subunit | -2.04 | 4.11E-10 | 91.39 | 375.97 |
| 28 | Dst | dystonin | -1.38 | 4.35E-10 | 619.00 | 1610.00 |
| 29 | Palld | palladin, cytoskeletal associated protein | -1.81 | 5.07E-10 | 79.35 | 279.23 |
| 30 | Cyp2c40 | cytochrome P450, family 2, subfamily c, polypeptide 40 | -2.29 | 5.07E-10 | 394.40 | 1926.57 |
| 31 | Cyp2c69 | cytochrome P450, family 2, subfamily c, polypeptide 69 | -2.88 | 5.60E-10 | 133.27 | 984.57 |
| 32 | Creb3l2 | cAMP responsive element binding protein 3-like 2 | -1.37 | 5.71E-10 | 191.77 | 497.53 |
| 33 | Csrp3 | cysteine and glycine-rich protein 3 | 1.40 | 6.31E-10 | 584.40 | 222.17 |
| 34 | Gsta1 | glutathione S-transferase, alpha 1 (Ya) | -3.68 | 7.84E-10 | 58.03 | 743.10 |
| 35 | Map3k13 | mitogen-activated protein kinase kinase kinase 13 | -1.94 | 8.68E-10 | 86.56 | 329.40 |
| 36 | Gpnmb | glycoprotein (transmembrane) nmb | -8.83 | 8.68E-10 | 0.90 | 445.24 |
| 37 | Cep85l | centrosomal protein 85-like | -2.25 | 8.75E-10 | 39.47 | 188.27 |
| 38 | Rasal2 | RAS protein activator like 2 | -1.58 | 9.85E-10 | 118.76 | 355.70 |
| 39 | Ankrd52 | ankyrin repeat domain 52 | -1.60 | 1.10E-09 | 134.47 | 409.50 |
| 40 | Apoa2 | apolipoprotein A-II | 1.45 | 1.10E-09 | 167033.33 | 61193.33 |
| 41 | Serpina7 | serine (or cysteine) peptidase inhibitor | 2.03 | 1.51E-09 | 873.33 | 213.03 |
| 42 | Lcor | ligand dependent nuclear receptor corepressor | -2.43 | 1.94E-09 | 37.14 | 198.53 |
| 43 | Dhrs9 | dehydrogenase/reductase (SDR family) member 9 | -2.01 | 2.03E-09 | 36.17 | 146.47 |
| 44 | Usp9x | ubiquitin specific peptidase 9, X chromosome | -1.45 | 2.52E-09 | 667.37 | 1825.67 |
| 45 | Ppara | peroxisome proliferator activated receptor alpha | -1.71 | 2.92E-09 | 404.60 | 1323.47 |
| 46 | Tstd1 | thiosulfate sulfurtransferase (rhodanese)-like domain containing 1 | 1.37 | 3.48E-09 | 431.60 | 166.90 |
| 47 | Yod1 | YOD1 OTU deubiquitinating enzyme 1 homologue (S. cerevisiae) | -1.75 | 4.14E-09 | 59.70 | 200.63 |
| 48 | Pik3ap1 | phosphoinositide-3-kinase adaptor protein 1 | -1.34 | 4.92E-09 | 201.33 | 511.87 |
| 49 | Btbd7 | BTB (POZ) domain containing 7 | -2.14 | 4.95E-09 | 38.78 | 170.90 |
| 50 | Per1 | period circadian clock 1 | -1.87 | 5.35E-09 | 86.20 | 319.67 |
| fdr false discovery rate | | | | | | |
