# Supplementary material for: Beyond detoxification: a role for mouse mEH in the hepatic metabolism of endogenous lipids
Source: Arch Toxicol. 2017 Oct 3;91(11):3571–85. doi: 10.1007/s00204-017-2060-4 (PMC5696502; doi:10.1007/s00204-017-2060-4)
Supplement: Supplementary file 5 — Supplementary material 5 (PPTX 70 kb) [file 204_2017_2060_MOESM5_ESM.pptx]

## Slide 1
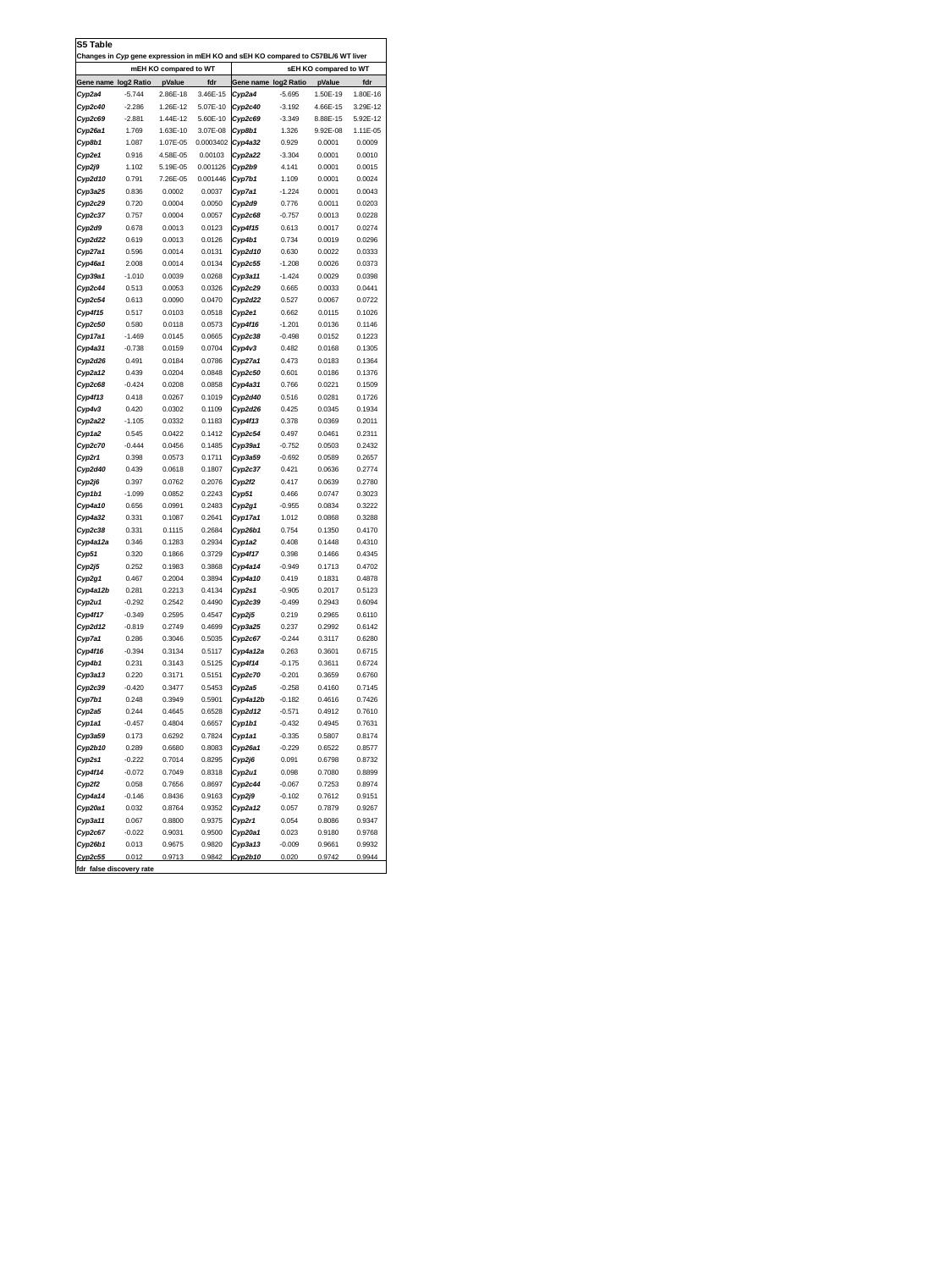

| S5 Table | | | | | | | |
| --- | --- | --- | --- | --- | --- | --- | --- |
| Changes in Cyp gene expression in mEH KO and sEH KO compared to C57BL/6 WT liver | | | | | | | |
| | mEH KO compared to WT | | | | sEH KO compared to WT | | |
| Gene name | log2 Ratio | pValue | fdr | Gene name | log2 Ratio | pValue | fdr |
| Cyp2a4 | -5.744 | 2.86E-18 | 3.46E-15 | Cyp2a4 | -5.695 | 1.50E-19 | 1.80E-16 |
| Cyp2c40 | -2.286 | 1.26E-12 | 5.07E-10 | Cyp2c40 | -3.192 | 4.66E-15 | 3.29E-12 |
| Cyp2c69 | -2.881 | 1.44E-12 | 5.60E-10 | Cyp2c69 | -3.349 | 8.88E-15 | 5.92E-12 |
| Cyp26a1 | 1.769 | 1.63E-10 | 3.07E-08 | Cyp8b1 | 1.326 | 9.92E-08 | 1.11E-05 |
| Cyp8b1 | 1.087 | 1.07E-05 | 0.0003402 | Cyp4a32 | 0.929 | 0.0001 | 0.0009 |
| Cyp2e1 | 0.916 | 4.58E-05 | 0.00103 | Cyp2a22 | -3.304 | 0.0001 | 0.0010 |
| Cyp2j9 | 1.102 | 5.19E-05 | 0.001126 | Cyp2b9 | 4.141 | 0.0001 | 0.0015 |
| Cyp2d10 | 0.791 | 7.26E-05 | 0.001446 | Cyp7b1 | 1.109 | 0.0001 | 0.0024 |
| Cyp3a25 | 0.836 | 0.0002 | 0.0037 | Cyp7a1 | -1.224 | 0.0001 | 0.0043 |
| Cyp2c29 | 0.720 | 0.0004 | 0.0050 | Cyp2d9 | 0.776 | 0.0011 | 0.0203 |
| Cyp2c37 | 0.757 | 0.0004 | 0.0057 | Cyp2c68 | -0.757 | 0.0013 | 0.0228 |
| Cyp2d9 | 0.678 | 0.0013 | 0.0123 | Cyp4f15 | 0.613 | 0.0017 | 0.0274 |
| Cyp2d22 | 0.619 | 0.0013 | 0.0126 | Cyp4b1 | 0.734 | 0.0019 | 0.0296 |
| Cyp27a1 | 0.596 | 0.0014 | 0.0131 | Cyp2d10 | 0.630 | 0.0022 | 0.0333 |
| Cyp46a1 | 2.008 | 0.0014 | 0.0134 | Cyp2c55 | -1.208 | 0.0026 | 0.0373 |
| Cyp39a1 | -1.010 | 0.0039 | 0.0268 | Cyp3a11 | -1.424 | 0.0029 | 0.0398 |
| Cyp2c44 | 0.513 | 0.0053 | 0.0326 | Cyp2c29 | 0.665 | 0.0033 | 0.0441 |
| Cyp2c54 | 0.613 | 0.0090 | 0.0470 | Cyp2d22 | 0.527 | 0.0067 | 0.0722 |
| Cyp4f15 | 0.517 | 0.0103 | 0.0518 | Cyp2e1 | 0.662 | 0.0115 | 0.1026 |
| Cyp2c50 | 0.580 | 0.0118 | 0.0573 | Cyp4f16 | -1.201 | 0.0136 | 0.1146 |
| Cyp17a1 | -1.469 | 0.0145 | 0.0665 | Cyp2c38 | -0.498 | 0.0152 | 0.1223 |
| Cyp4a31 | -0.738 | 0.0159 | 0.0704 | Cyp4v3 | 0.482 | 0.0168 | 0.1305 |
| Cyp2d26 | 0.491 | 0.0184 | 0.0786 | Cyp27a1 | 0.473 | 0.0183 | 0.1364 |
| Cyp2a12 | 0.439 | 0.0204 | 0.0848 | Cyp2c50 | 0.601 | 0.0186 | 0.1376 |
| Cyp2c68 | -0.424 | 0.0208 | 0.0858 | Cyp4a31 | 0.766 | 0.0221 | 0.1509 |
| Cyp4f13 | 0.418 | 0.0267 | 0.1019 | Cyp2d40 | 0.516 | 0.0281 | 0.1726 |
| Cyp4v3 | 0.420 | 0.0302 | 0.1109 | Cyp2d26 | 0.425 | 0.0345 | 0.1934 |
| Cyp2a22 | -1.105 | 0.0332 | 0.1183 | Cyp4f13 | 0.378 | 0.0369 | 0.2011 |
| Cyp1a2 | 0.545 | 0.0422 | 0.1412 | Cyp2c54 | 0.497 | 0.0461 | 0.2311 |
| Cyp2c70 | -0.444 | 0.0456 | 0.1485 | Cyp39a1 | -0.752 | 0.0503 | 0.2432 |
| Cyp2r1 | 0.398 | 0.0573 | 0.1711 | Cyp3a59 | -0.692 | 0.0589 | 0.2657 |
| Cyp2d40 | 0.439 | 0.0618 | 0.1807 | Cyp2c37 | 0.421 | 0.0636 | 0.2774 |
| Cyp2j6 | 0.397 | 0.0762 | 0.2076 | Cyp2f2 | 0.417 | 0.0639 | 0.2780 |
| Cyp1b1 | -1.099 | 0.0852 | 0.2243 | Cyp51 | 0.466 | 0.0747 | 0.3023 |
| Cyp4a10 | 0.656 | 0.0991 | 0.2483 | Cyp2g1 | -0.955 | 0.0834 | 0.3222 |
| Cyp4a32 | 0.331 | 0.1087 | 0.2641 | Cyp17a1 | 1.012 | 0.0868 | 0.3288 |
| Cyp2c38 | 0.331 | 0.1115 | 0.2684 | Cyp26b1 | 0.754 | 0.1350 | 0.4170 |
| Cyp4a12a | 0.346 | 0.1283 | 0.2934 | Cyp1a2 | 0.408 | 0.1448 | 0.4310 |
| Cyp51 | 0.320 | 0.1866 | 0.3729 | Cyp4f17 | 0.398 | 0.1466 | 0.4345 |
| Cyp2j5 | 0.252 | 0.1983 | 0.3868 | Cyp4a14 | -0.949 | 0.1713 | 0.4702 |
| Cyp2g1 | 0.467 | 0.2004 | 0.3894 | Cyp4a10 | 0.419 | 0.1831 | 0.4878 |
| Cyp4a12b | 0.281 | 0.2213 | 0.4134 | Cyp2s1 | -0.905 | 0.2017 | 0.5123 |
| Cyp2u1 | -0.292 | 0.2542 | 0.4490 | Cyp2c39 | -0.499 | 0.2943 | 0.6094 |
| Cyp4f17 | -0.349 | 0.2595 | 0.4547 | Cyp2j5 | 0.219 | 0.2965 | 0.6110 |
| Cyp2d12 | -0.819 | 0.2749 | 0.4699 | Cyp3a25 | 0.237 | 0.2992 | 0.6142 |
| Cyp7a1 | 0.286 | 0.3046 | 0.5035 | Cyp2c67 | -0.244 | 0.3117 | 0.6280 |
| Cyp4f16 | -0.394 | 0.3134 | 0.5117 | Cyp4a12a | 0.263 | 0.3601 | 0.6715 |
| Cyp4b1 | 0.231 | 0.3143 | 0.5125 | Cyp4f14 | -0.175 | 0.3611 | 0.6724 |
| Cyp3a13 | 0.220 | 0.3171 | 0.5151 | Cyp2c70 | -0.201 | 0.3659 | 0.6760 |
| Cyp2c39 | -0.420 | 0.3477 | 0.5453 | Cyp2a5 | -0.258 | 0.4160 | 0.7145 |
| Cyp7b1 | 0.248 | 0.3949 | 0.5901 | Cyp4a12b | -0.182 | 0.4616 | 0.7426 |
| Cyp2a5 | 0.244 | 0.4645 | 0.6528 | Cyp2d12 | -0.571 | 0.4912 | 0.7610 |
| Cyp1a1 | -0.457 | 0.4804 | 0.6657 | Cyp1b1 | -0.432 | 0.4945 | 0.7631 |
| Cyp3a59 | 0.173 | 0.6292 | 0.7824 | Cyp1a1 | -0.335 | 0.5807 | 0.8174 |
| Cyp2b10 | 0.289 | 0.6680 | 0.8083 | Cyp26a1 | -0.229 | 0.6522 | 0.8577 |
| Cyp2s1 | -0.222 | 0.7014 | 0.8295 | Cyp2j6 | 0.091 | 0.6798 | 0.8732 |
| Cyp4f14 | -0.072 | 0.7049 | 0.8318 | Cyp2u1 | 0.098 | 0.7080 | 0.8899 |
| Cyp2f2 | 0.058 | 0.7656 | 0.8697 | Cyp2c44 | -0.067 | 0.7253 | 0.8974 |
| Cyp4a14 | -0.146 | 0.8436 | 0.9163 | Cyp2j9 | -0.102 | 0.7612 | 0.9151 |
| Cyp20a1 | 0.032 | 0.8764 | 0.9352 | Cyp2a12 | 0.057 | 0.7879 | 0.9267 |
| Cyp3a11 | 0.067 | 0.8800 | 0.9375 | Cyp2r1 | 0.054 | 0.8086 | 0.9347 |
| Cyp2c67 | -0.022 | 0.9031 | 0.9500 | Cyp20a1 | 0.023 | 0.9180 | 0.9768 |
| Cyp26b1 | 0.013 | 0.9675 | 0.9820 | Cyp3a13 | -0.009 | 0.9661 | 0.9932 |
| Cyp2c55 | 0.012 | 0.9713 | 0.9842 | Cyp2b10 | 0.020 | 0.9742 | 0.9944 |
| fdr false discovery rate | | | | | | | |
